# Taxonomy, Diet, and Developmental Stage Contribute to the Structuring of Gut-Associated Bacterial Communities in Tephritid Pest Species

**DOI:** 10.3389/fmicb.2019.02004

**Published:** 2019-08-29

**Authors:** Antonios A. Augustinos, George Tsiamis, Carlos Cáceres, Adly M. M. Abd-Alla, Kostas Bourtzis

**Affiliations:** ^1^Insect Pest Control Laboratory, Joint FAO/IAEA Division of Nuclear Techniques in Food and Agriculture, Vienna, Austria; ^2^Department of Environmental Engineering, University of Patras, Agrinio, Greece

**Keywords:** *Anastrepha*, *Bactrocera*, sterile insect technique, pest control, laboratory domestication, 16S rRNA, amplicon sequencing

## Abstract

Insect-symbiont interactions are receiving much attention in the last years. Symbiotic communities have been found to influence a variety of parameters regarding their host physiology and fitness. Gut symbiotic communities can be dynamic, changing through time and developmental stage. Whether these changes represent real differential needs and preferential relationships has not been addressed yet. In this study, we characterized the structure of symbiotic communities of five laboratory populations that represent five Tephritidae species that are targets for pest control management through the sterile insect technique (SIT), namely *Bactrocera oleae, Anastrepha grandis, Anastrepha ludens*, and two morphotypes of *Anastrepha fraterculus* (sp.1 and the Andean lineage). These populations are under artificial or semi artificial rearing conditions and their characterization was performed for different developmental stages and age. Our results demonstrate the presence of a symbiotic community comprising mainly from different Enterobacteriaceae genera. These communities are dynamic across developmental stages, although not highly variable, and appear to have a species-specific profile. Additional factors may contribute to the observed structuring, including diet, rearing practices, and the degree of domestication. Comparison of these results with those derived from natural populations could shed light to changes occurring in the symbiotic level during domestication of Tephritidae populations. Further studies will elucidate whether the changes are associated with modification of the behavior in laboratory strains and assess their effects in the quality of the mass rearing insects. This could be beneficial for improving environmentally friendly, species-specific, pest control methods, such as the SIT.

## Introduction

Tephritidae is a family of Diptera harboring more than 500 genera and 4600 species (Norrbom, 2004; Virgilio et al., 2009, 2014). Among tephritid genera, *Anastrepha, Bactrocera*, *Ceratitis*, *Dacus, Rhagoletis*, and *Zeugodacus* include frugivorous species, with around 100 of them being agricultural pests of economic importance (White and Elson-Harris, 1992; Norrbom, 2004; Virgilio et al., 2009, 2014). Economic damage is due to the oviposition of eggs in the mesocarp, the subsequent reduction of quality of the fruit due to the punctures made during oviposition, and the loss of production due to larval feeding (White and Elson-Harris, 1992).

Among the methods used against insect pests, environmentally friendly control methods have received attention in the last decades, enhanced by the documented environmental and health concerns associated with the extensive use of pesticides. In this respect, area-wide integrated pest management (AW-IPM) utilizes different approaches that synergistically can drastically suppress or locally eliminate the pest population. A major component of IPM for a variety of pests is the sterile insect technique (SIT). SIT is based on the release of sterile insects, preferentially only males, that can mate with the female flies of the natural population, leading to infertile crosses and, ultimately, reduction in population size (Dyck et al., 2005).

One of the previously underestimated factors that can affect the behavior of laboratory strains is their symbiotic communities. Recent studies have shown that symbionts, especially gut microbiota, can affect different parameters that are important for the insects’ physiology and life history traits (Bourtzis and Miller, 2003, 2006, 2009; Ben-Yosef et al., 2008, 2014; Douglas, 2009, 2015; Zchori-Fein and Bourtzis, 2011; Engel and Moran, 2013; Minard et al., 2013). Within Tephritidae, many studies have been performed in the Mediterranean fruit fly, *Ceratitis capitata*, providing evidence for the structure of the gut symbiotic community of both natural and laboratory populations (Behar et al., 2008; Lauzon et al., 2009; Ben Ami et al., 2010; Aharon et al., 2012; Augustinos et al., 2015; Malacrino et al., 2018) and the impact of specific bacteria used as probiotics or alternative protein sources (Niyazi et al., 2004; Behar et al., 2008; Ben-Yosef et al., 2008; Ben Ami et al., 2010; Gavriel et al., 2011; Aharon et al., 2012; Hamden et al., 2013; Augustinos et al., 2015; Kyritsis et al., 2017). Besides medfly, studies have been performed in the olive fruit fly, *Bactrocera oleae* (Capuzzo, 2005; Sacchetti et al., 2008; Estes et al., 2012b; Savio et al., 2012; Ben-Yosef et al., 2014; Koskinioti et al., 2019) and to a few other tephritid species, mainly *Bactrocera* (Wang et al., 2011; Prabhakar et al., 2013; Thaochan et al., 2013; Pramanik et al., 2014; Wang A. et al., 2014; Wang H. et al., 2014; Andongma et al., 2015; Hadapad et al., 2015; Deutscher et al., 2017, 2018; Gujjar et al., 2017; Yong et al., 2017; Akami et al., 2019). Fewer studies are available in *Anastrepha* species, focusing on *A. ludens* (Kuzina et al., 2001) and more recently, in wild samples of four different species (Ventura et al., 2018).

The primary target of the insect pest control laboratory (IPCL) of the Joint FAO/IAEA Division of Nuclear Techniques in Food and Agriculture (Seibersdorf, Austria) is to develop the SIT as a component of AW-IPM projects against different insect pest species. Many problems arise from the suboptimal conditions regarding the initial steps of laboratory domestication as well as mass rearing, including artificial diet and artificial substrates for oviposition. In some cases, such constraints can modify the behavior of laboratory strains, affecting therefore the efficiency of SIT (Dyck et al., 2005; Estes et al., 2012b; Rempoulakis et al., 2018). The changes that can happen in the symbiotic communities of laboratory populations due to long established domestication and artificial conditions are worth investigating.

In this study, the gut symbiotic communities of five selected tephritid laboratory populations were analyzed using *16S* rRNA gene-based NGS approaches: two populations representing two morphotypes of the *Anastrepha fraterculus* species complex, *Anastrepha ludens*, *Anastrepha grandis*, and *Bactrocera oleae*. *A. fraterculus* and *A. ludens* are well adapted in artificial conditions, while a totally artificial diet has not yet been achieved for *A. grandis.* Regarding *B. oleae*, its artificial rearing can be considered far from optimal, since many laboratories are either suffering from occasional collapses of their colonies or must follow a semi-artificial rearing, using olive fruit as an oviposition substrate. Whether differences in the performance among laboratory-adapted populations of a given species could be attributed, at least partially, to the changes of the structure of their symbiotic communities has not yet been addressed. Aiming to “dissect” factors that may contribute to the structuring of gut symbiotic communities, samples representing different developmental stages, age, and sex, were collected and analyzed.

## Materials and Methods

### Rearing Conditions, Sample Collection, and Preparation

The laboratory populations studied are currently colonized in IPCL and include *A. fraterculus* sp. 1 (Vera et al., 2006), *A. fraterculus* (Andean lineage) (Hernández-Ortiz et al., 2015), *Anastrepha grandis* (Hallman et al., 2017), *A. ludens* (Eskafi, 1988), and *B. oleae* (Ahmad et al., 2016). The full description of these strains, along with their rearing conditions is summarized in Table 1.

**TABLE 1:** Strains used, their origin, and rearing conditions.

**Species**	**Symbol**	**Generations in IPCL**	**Adult diet**	**Larval diet^2^**	**Humidity (%)**	**Temperature (°C)**	**Photoperiod**
*Anastrepha fraterculus* sp. 1	Af1	84	1:3 (yeast: sugar)	Carrot	65	25	14 light: 10 dark
*Anastrepha fraterculus* (Andean lineage)	AfA	10	1:3 (yeast: sugar)	Carrot	65	25	14 light: 10 dark
*Anastrepha grandis*	Agr	7	1:3 (yeast: sugar)	Specialized^3^	65	25	14 light: 1 dark
*Anastrepha ludens*	Alu	7	1:3 (yeast: sugar)	Carrot	65	25	14 light: 10 dark
*Bactrocera oleae*	Bol	117	Specialized^1^	Specialized^4^	65	25	14 light: 10 dark

*^1^The specialized adult diet for *B. oleae* consists of 75% sugar, 19% hydrolyzed yeast, and 6% egg yolk powder without antibiotics. ^2^The typical carrot diet consists of 7% brewer’s yeast, 0.25% sodium benzoate, 0.2% methylparaben, 0.8% (v/w) HCl, 15% carrot powder, and all dissolved in water. ^3^*Anastrepha grandis* larvae diet is semi-artificial, since young larvae are transferred and reared in zucchini. ^4^*B. oleae* larvae diet consists of 52.08 ml water, 2.08 ml virgin olive oil, 0.78 ml Tween 80 (emulsifier), 0.05 gr potassium sorbate, 0.21 gr methylparaben, 2.08 gr sugar, 7.81 gr brewer’s yeast, 3.13 gr soy hydrolyzed, 3.13 ml HCl (2N) and 28.65 gr cellulose powder.*

### Gut Collections and Dissections

Guts were collected for each of the strains from 3rd instar larvae (L), 1-day-old males (1D_M) and females (1D_F) that had not been fed, 5–10-days old males (10D_M) and females (10D_F), and 15–20-days old males (15D_M) and females (15D_F). Samples of guts were collected in batches of five individuals in three biological replicates (a total of 15 individuals). Prior to dissections, flies were immobilized/anesthetized at 4°C and disinfected (surface-sterilized) through dipping in 70% ethanol and subsequently were kept in sterile phosphate-buffered saline of 1(×) concentration (1× PBS). Dissections were also performed in sterile 1x PBS under aseptic conditions. Samples were stored in −20°C until DNA extraction. The sampling scheme is presented in Supplementary Table S1.

### DNA Extraction and 16S rRNA Gene Amplicon Library Preparation and Sequencing

Prior to extraction, guts were homogenized in liquid nitrogen using sterile polypropylene pestles. Subsequently, DNA was extracted using the DNeasy Blood and Tissue kit (Qiagen) following manufacturer’s instructions for total DNA purification from animal tissues. Negative controls were included in DNA extraction. DNA quality and quantity were measured using the NanoDrop 1000 Spectrophotometer (Thermo scientific). A total of 105 samples were analyzed using 454 NGS for the 16S *rRNA* gene with the following primers, targeting the V6-V8 hypervariable region: 926F (AAA CTY AAA KGA ATT GAC GRC GG) and 1392R (ACG GGC GGT GTG TRC) (Rinke et al., 2013). PCRs were performed by Macrogen^1^, after linking the primer with decamer multiplex identifier (MID) sequences and adaptors for the GS FLX Titanium Chemistry to facilitate library multiplexing in the 454-sequencing system. In brief PCR was performed under the following conditions: 94°C for 3 min followed by 32 cycles of 94°C for 30 s; 55°C for 40 s and 72°C for 1 min; and a final elongation step at 72°C for 5 min. Following PCR, all amplicon products from different samples were mixed in equal concentrations and purified using Agencourt Ampure beads (Agencourt Bioscience Corporation, United States). Samples were sequenced utilizing Roche 454 FLX titanium instruments and reagents and following manufacturer’s guidelines.

### Bioinformatics and Data Analysis

Sequences were analyzed and processed using the QIIME package (Caporaso et al., 2010). Briefly, the QIIME pipeline takes all sequences from a single pyrosequencing run and assigns sample IDs using a mapping file and the barcode assigned to each sample. Sequences were excluded from the analysis if they were <200 bp in length, had a quality score of <25, contained ambiguous characters or did not contain the primer sequence. Sickle version 1.200 was used to trim reads based on quality: any reads with a window quality score of less than 20, or which were less than 10 bp long after trimming, were discarded (Joshi and Fass, 2011). BayesHammer was used to correct reads based on quality (Nikolenko et al., 2013). Chimeras were detected and omitted using the program UCHIME with the QIIME-compatible version of the SILVA111 release database (Quast et al., 2013). The 16S *rRNA* gene sequences were clustered using the usearch algorithm (Edgar, 2010) and assigned to operational taxonomic units (OTUs) with 97% similarity. Representative sequences from each OTU were aligned with Pynast (Caporaso et al., 2010) against the Greengenes core reference alignment (version gg_12_10). Taxonomy was assigned using the SILVA 16S *rRNA* gene database. The *16S* rRNA gene sequences reported in this study have been deposited in NCBI under Bioproject number PRJNA525967.

α-diversity indices, as well as indices depicting the population structure, were calculated with the QIIME pipeline (Caporaso et al., 2010) based on the rarefied OTU table at a depth of 5,000 sequences/sample (observed species, PD whole tree, chao1 and simpson reciprocal). Variation between replicates was low since all replicates were a pool of 5 tissues. Inter-sample diversity was calculated using Bray-Curtis distances, and principal coordinate analysis (PCoA) was performed on the resulting distance matrix. These calculations and those for alpha diversity were performed in QIIME version 1.9.1. ANOVA and Tukey-Kramer *post hoc* tests were employed to detect statistical differences (Edgar, 2010). Overall similarities in bacterial community structures were shown using the unconstrained ordination technique, principal coordinate analysis, multidimensional scaling (MDS) analysis, and the multidimensional plots as implemented in PRIMER version 6+ (Anderson, 2001). Permutational multivariate analysis of variance (PERMANOVA) analyses were applied to Bray-Curtis similarity matrices to compute similarities between groups. Differences in community structure were viewed using the constrained ordination technique CAP (canonical analysis of principal coordinates), using the CAP classification success rate and CAP trace_Q_m__’__HQ_m_ statistics, and were performed with 9999 permutations within PRIMER version 6+ (Anderson and Willis, 2003). CAP analysis was performed using the Bray-Curtis similarity matrices.

### Checking for Missing Tenants

Curated 16S *rRNA* gene sequences from the Greengenes and SILVA databases were retrieved, corresponding to genera known to be part of tephritid symbiotic communities. Sequences were aligned, and the respective fragment amplified with 926F – 1392R primers was selected *in silico*. These sequences were incorporated in our data set and were analyzed through the QIIME pipeline as described before.

## Results

### 16S rRNA Gene Pyrosequencing

A total of 2,254,978 raw reads were obtained from the 454 processing; 1,544,398 reads passed the filters applied through QIIME for non-eukaryotic sequences, with an average of 14,890 reads/sample. After grouping the three replicates, number of reads per sample ranged from 1351 to 93543 (see Table 2). Analysis of alpha diversity measures and respective rarefaction curves show that library coverage was adequate to capture the whole diversity of the gut symbiotic communities for almost all samples (Supplementary Figure S1).

**TABLE 2:** Number of reads and bacterial diversity indices of all samples.

**Sample^1^**	**Number of reads^2^**	**Number of OTUs**	**Species richness indices**		**Species diversity indices**	
			**Chao1**	**Menhinick**	**Shannon**	**Simpson**
Af1_L_G	450.33	23,33 ± 7.69	28.61 ± 10.76	1.266 ± 0.07	3.154 ± 0.33	0.823 ± 0.02
Af1_1D_M_G	997.33	33.33 ± 6.06	35.86 ± 5.32	1.225 ± 0.21	4.040 ± 0.23	0.910 ± 0.02
Af1_1D_F_G	1608	18.00 ± 1.73	29.53 ± 9.70	1.223 ± 0.54	2.292 ± 0.80	0.633 ± 0.19
Af_5D_M_G	10904	26.00 ± 9.45	34.66 ± 11.89	0.251 ± 0.08	1.877 ± 0.37	0.602 ± 0.05
Af1_5D_F_G	7260.67	16.66 ± 4.37	19.16 ± 5.80	0.204 ± 0.06	1.903 ± 0.64	0.575 ± 0.17
Af1_15D_M_G	13555.33	9.33 ± 0.33	9.66 ± 0.33	0.094 ± 0.02	1.621 ± 0.34	0.556 ± 0.11
Af1_15D_F_G	24513.33	10.00 ± 0.58	10.6 ± 0.66	0.066 ± 0.01	0.896 ± 0.29	0.280 ± 0.11
AfA_L_G	4614.33	18.00 ± 6.60	19.77 ± 7.80	0.333 ± 0.10	1.108 ± 0.17	0.374 ± 0.05
AfA_1D_M_G	2677.67	34.33 ± 6.43	39.68 ± 4.04	0.881 ± 0.30	2.930 ± 0.61	0.742 ± 0.09
AfA_1D_F_G	11633.67	27.33 ± 6.08	29.58 ± 6.69	0.409 ± 0.17	1.604 ± 0.50	0.490 ± 0.12
AfA_5D_M_G	12361.33	15.66 ± 5.86	16.77 ± 6.76	0.135 ± 0.03	0.970 ± 0.24	0.288 ± 0.10
AfA_5D_F_G	7634.33	18.33 ± 3.57	19.33 ± 3.34	0.211 ± 0.02	1.797 ± 0.28	0.583 ± 0.06
AfA_15D_M_G	16545.00	24.33 ± 6.06	26.44 ± 6.58	0.208 ± 0.02	1.303 ± 0.10	0.404 ± 0.01
AfA_15D_F_G	6400.33	12.33 ± 1.09	16.33 ± 3.54	0.631 ± 0.39	2.029 ± 0.93	0.519 ± 0.18
Alu_L_G	18153.67	14.33 ± 0.98	16.16 ± 0.49	0.103 ± 0.01	0.546 ± 0.26	0.206 ± 0.13
Alu_1D_M_G	30594.67	28.33 ± 2.99	33.66 ± 3.14	0.531 ± 0.20	1.716 ± 0.81	0.421 ± 0.18
Alu_1D_F_G	30072.67	31.66 ± 5.19	39.40 ± 7.65	0.760 ± 0.24	2.135 ± 0.75	0.545 ± 0.19
Alu_5D_M_G	18493.67	18.00 ± 2.87	20.08 ± 2.42	0.132 ± 0.04	0.957 ± 0.55	0.282 ± 0.17
Alu_5D_F_G	22066.67	15.66 ± 1.08	16.05 ± 1.05	0.088 ± 0.01	0.437 ± 0.06	0.111 ± 0.01
Alu_15D_M_G	16340	20.00 ± 0.94	22.64 ± 2.65	0.291 ± 0.06	1.273 ± 0.39	0.391 ± 0.13
Alu_15D_F_G	20527.67	29.33 ± 0.27	32.44 ± 2.81	0.276 ± 0.03	2.341 ± 0.27	0.679 ± 0.07
Agr_L_G	19733.33	39.66 ± 5.82	48.50 ± 9.57	0.294 ± 0.03	1.515 ± 0.39	0.430 ± 0.13
Agr_1D_M_G	11965.33	14.33 ± 6.43	17.00 ± 7.07	0.098 ± 0.05	0.247 ± 0.19	0.062 ± 0.05
Agr_1D_F_G	8712.33	9.00 ± 1.70	11.33 ± 2.84	0.053 ± 0.01	0.219 ± 0.16	0.088 ± 0.07
Agr_5D_M_G	23014.33	30.66 ± 1.65	31.77 ± 2.45	0.240 ± 0.03	1.699 ± 0.25	0.459 ± 0.07
Agr_5D_F_G	31314.33	39.33 ± 1.44	47.04 ± 4.48	0.291 ± 0.03	1.512 ± 0.49	0.394 ± 0.13
Agr_15D_M_G	7764.67	29.66 ± 1.66	33.83 ± 3.89	0.242 ± 0.02	2.031 ± 0.12	0.605 ± 0.04
Agr_15D_F_G	12023.33	30.00 ± 1.63	31.44 ± 1.69	0.219 ± 0.03	1.943 ± 0.59	0.537 ± 0.15
Bol_L_G	15181.33	5.00 ± 1.70	7.00 ± 2.94	0.147 ± 0.07	0.146 ± 0.10	0.043 ± 0.03
Bol_1D_M_G	11604	9.33 ± 0.27	12.50 ± 1.08	0.305 ± 0.10	0.739 ± 0.18	0.228 ± 0.05
Bol_1D_F_G	22276.67	10.66 ± 2.13	10.50 ± 1.65	0.047 ± 0.01	0.013 ± 0.01	0.002 ± 0.01
Bol_5D_M_G	26805	12.66 ± 3.57	14.27 ± 4.20	0.081 ± 0.02	0.227 ± 0.05	0.057 ± 0.01
Bol_5D_F_G	22400.67	14.00 ± 2.36	14.33 ± 2.37	0.095 ± 0.02	0.697 ± 0.27	0.219 ± 0.08
Bol_15D_M_G	649.67	3.33 ± 1.09	3.66 ± 1.36	0.143 ± 0.05	0.724 ± 0.12	0.311 ± 0.08
Bol_15D_F_G	23949.67	8.33 ± 3.67	9.50 ± 4.42	0.069 ± 0.01	0.933 ± 0.41	0.378 ± 0.16

*Af1, *A. fraterculus* sp. 1; AfA, *A. fraterculus* (Andean lineage); Agr, *A. grandis*; Alu, *A. ludens*; Bol, *B. oleae*; L, larvae; M: males; F, females; 1D, 1-day old, unfed; 5D, 5–10-days old; 15D, 15–20-days old; G, gut.*

### Structure of the Gut-Associated Bacterial Communities of the Five Laboratory Populations

The *Anastrepha fraterculus* Af1 laboratory population was mainly dominated by *Proteobacteria* in all samples, followed by *Firmicutes*, *Actinobacteria*, *Bacteroidetes*, and *Cyanobacteria* which were present at a low relative abundance (RA) (Supplementary Excel S1 and Supplementary Figure S2A). The most dominant class of *Proteobacteria* was Gammaproteobacteria with a RA up to 100% in some samples (Supplementary Excel S1 and Supplementary Figure S2B) with all abundant OTUs present in all replicates. It’s worth noting that the symbiotic community was rather dynamic with fluctuations in the species richness and the RA of different genera (Table 2, Supplementary Excel S1, and Supplementary Figure S2C). PCoA analysis indicated the formation of three clusters with the first two axes accounting for the 62.7% of the total variation (Supplementary Figure S3A), although intra-sample variability was also evident (Supplementary Figure S3B). Permanova analysis indicated that the clusters observed were statistically significant (*p* < 0.001) with the 3rd instar larvae constituting the first cluster, the 1-day old adults forming the second cluster, and the 5–10 day and 15–20 days old adults forming the third cluster.

*Proteobacteria* was the prevailing Phylum in all samples of the *A. fraterculus* (AfA) laboratory population followed by *Firmicutes*, *Actinobacteria*, *Bacteroidetes*, *Deinococcus*-*Thermus*, *Actinobacteria*, and *Aquificae* (Supplementary Excel S1 and Supplementary Figure S4A). Among *Proteobacteria*, the majority belonged to Gammaproteobacteria (Supplementary Excel S1 and Supplementary Figure S4B). The symbiotic community of this population was also dynamic with fluctuations in the species richness and the RA of different genera (Table 2, Supplementary Excel S1, and Supplementary Figure S4C). Interestingly, two members of the dominant family of Enterobacteriaceae, *Morganella* and *Enterobacter*, seem to have completely different patterns, with the first one being a major component of the symbiotic communities of larvae and 1-day old adults and almost undetectable in the older adult stages, while the second one being almost undetectable in L and 1-day old females and becoming the most (or nearly the most) abundant genus in older flies (Supplementary Excel S1 and Supplementary Figure S4C). PCoA analysis indicated the presence of two distinct clusters, with the first two axes describing the 65.4% of the total variation (Supplementary Figure S5). Permanova analysis verified that the 3rd instar larvae and the 1-day old flies formed a distinct group, separate from the 5 to 10 days and 15 to 20 days old flies forming the second group (*p* < 0.001).

*Proteobacteria* dominated all samples of the *Anastrepha ludens* (Alu) laboratory population, followed by *Firmicutes* 1-day old males (Supplementary Excel S1 and Supplementary Figure S6A). Gammaproteobacteria was the most dominant class with Bacilli being also an abundant component of the symbiotic community, which was characterized by fluctuations in species richness and RA during development (Table 2, Supplementary Excel S1, and Supplementary Figure S6B). In Gammaproteobacteria, members of the family Enterobacteriaceae, such as *Providencia, Enterobacter*, and *Klebsiella*, were the most abundant (Supplementary Excel S1 and Supplementary Figure S6C). The first two axes of the PCoA accounted for the 57.6% of the observed variance (Supplementary Figure S7A). Permanova analysis indicated that the developmental stage and age of the adults had a significant role in the formation of the bacterial profile of the gastrointestinal tract (*p* < 0.002) (Supplementary Figure S7B). More specifically, the *A. ludens* larval gut bacterial profile was statistically different only from the 1-day and 15 days old flies (*p* < 0.033, and *p* < 0.01, respectively). All other combinations were not statistically different.

*Proteobacteria* was the prevailing phylum in the laboratory population of *Anastrepha grandis* (Agr), followed by *Bacteroidetes, Firmicutes*, and *Actinobacteria*, although not present in RA higher than 1% in all samples (Supplementary Excel S1 and Supplementary Figure S8A). Gammaproteobacteria was the dominant class in this symbiotic community, which was characterized by changes in the species richness and RA during development (Table 2, Supplementary Excel S1, and Supplementary Figure S8B). Members of the family Enterobacteriaceae seemed to play a major role with *Providencia* being quite abundant followed, in some stages, by *Klebsiella*, *Morganella*, and *Enterobacter* (Supplementary Excel S1 and Supplementary Figure S8C). These differences are displayed in PCoA analysis, which captured 71.9% of the observed variance (Supplementary Figure S9A). Permanova analysis indicated that developmental stage and age of adults were significant factors affecting the bacterial profile in the gastrointestinal tract (*p* < 0.002) (Supplementary Figure S9B). The 1-day old flies formed a group distinct from the 10 to 15 days and 15 to 20 days old flies but also from the 3rd instar larvae (*p* < 0.003, *p* < 0.002, and *p* < 0.011, respectively).

The *Bactrocera oleae* (Bol) laboratory population was also dominated by *Proteobacteria* ranging between RA 85% and 100% in the different stages. *Bacteroidetes*, *Firmicutes*, and *Actinobacteria* followed at rather low RA (less than 1% in the majority of the samples) (Supplementary Excel S1 and Supplementary Figure S10A). The great majority of *Proteobacteria* belonged to Gammaproteobacteria (reaching up to 100% in some of the samples) (Supplementary Excel S1 and Supplementary Figure S10B). Interestingly, *Morganella* was a major component in all samples, since it ranged from 62 to 98%, while *Providencia, Acinetobacter, Enterobacter*, and *Klebsiella* were also present at low RA (Supplementary Excel S1 and Supplementary Figure S10C). PCoA failed to provide a clear clustering of the samples based either on developmental stage, sex or age, although it captures 79.5% of the observed variance (Supplementary Figure S11). Based on Permanova analysis, adult age and the developmental stage of *B. oleae* did not play a significant role in the formation of the gut bacterial profile (*p* < 0.115), however, the 1-day old females were quite differentiated from all other samples.

### Comparison of the Gut-Associated Bacterial Communities of the Different Developmental Stages of the Five Laboratory Populations

Trying to “dissect” consistent similarities and differences among the laboratory populations of the different species, we excluded the differences that could derive from changes happening during development. To do so, samples of the same developmental stages of the different laboratory populations were compared.

During the larval stage, the five populations exhibited different levels of bacterial OTU diversity with Agr having the highest one (Table 2). PCoA analysis demonstrated that there was a significant difference among the symbiotic communities of the larval guts of the five laboratory populations, consistent with their taxonomy (*p* < 0.001) (Supplementary Figure S12). The gut symbiotic communities of the three Bol replicates cluster together, apart from other samples (*p* < 0.001). No statistically significant differences are evident among the larval stages of the *Anastrepha* species, although there is a tendency for clustering per species. The larval bacterial profile of the Af1 colony is not statistically different from that of the AfA colony (*p* < 0.104). Alu samples cluster together, and close to the remaining Agr and AfA samples (*p* < 0.09 and *p* < 0.099, respectively).

The symbiotic communities of 1-day old flies of Af1, AfA, and Alu, were more diverse than those of Agr and Bol (Table 2). PCoA analysis demonstrates that there are significant differences among the symbiotic communities of the 1-day old guts of the five colonies that can be attributed to their different origin (species, geographic origin) (Supplementary Figures S13A,B) (*p* < 0.001). Samples representing the gut symbiotic communities of Bol and Agr (*p* < 0.002) are clearly differentiated from each other and all other samples, while samples from the three remaining populations (Alu, AfA, and Af1) are not clearly differentiated from each other (*p* < 0.085).

The symbiotic communities of 5–10 days old flies of Agr were more diverse than those of all other populations (Table 2). *Morganella* was the most abundant symbiont in the Bol population but was either undetected or in RA <2% in all other samples. *Klebsiella* (Enterobacteriaceae) and *Streptococcus* (Streptococcaceae) were the main components of the Alu gut symbiotic community contributing together 99% and 65% of the RA for the females and males of this population, respectively. However, these genera were either undetected or found at very low RAs (less than 0.1%) in all other populations (Supplementary Excel S1). PCoA analysis demonstrates that there are significant differences among the symbiotic communities of the 5–10 days old adult guts of the five colonies that can be attributed to their different origin (species and geographic origin) and/or to the different rearing practices followed (*p* < 0.001) (Supplementary Figures S14A,B). The gut symbiotic communities of Bol and Alu are clearly differentiated from each other (*p* < 0.005) and from all others (*p* < 0.006 Bol/AfA, *p* < 0.005 Bol/Agr, *p* < 0.005 Bol/Af1, *p* < 0.01 Alu/AfA, *p* < 0.005 Alu/Agr, *p* < 0.004 Alu/Af1) while, Agr, AfA and Af1 samples cluster together (*p* < 0.412 AfA/Af1, *p* < 0.056 AfA/Agr, *p* < 0.076 Af1/Agr).

The overall symbiotic diversity of the 15–20 days old fly samples was low (Table 2). Clustering of the 15–20 days old samples is not as clear as in the previous developmental stages and ages. PCoA analysis demonstrated that the gut symbiotic communities of Bol cluster apart from all *Anastrepha* samples (Permanova; *p* < 0.001), while no obvious clustering was evident within *Anastrepha* samples (Supplementary Figures S15A,B).

### Analysis of Possible Factors Influencing the Structure of the Gut Microbiome

#### Species Effect Plus Phylogenetic Distance and/or Rearing Conditions

**FIGURE 1:**
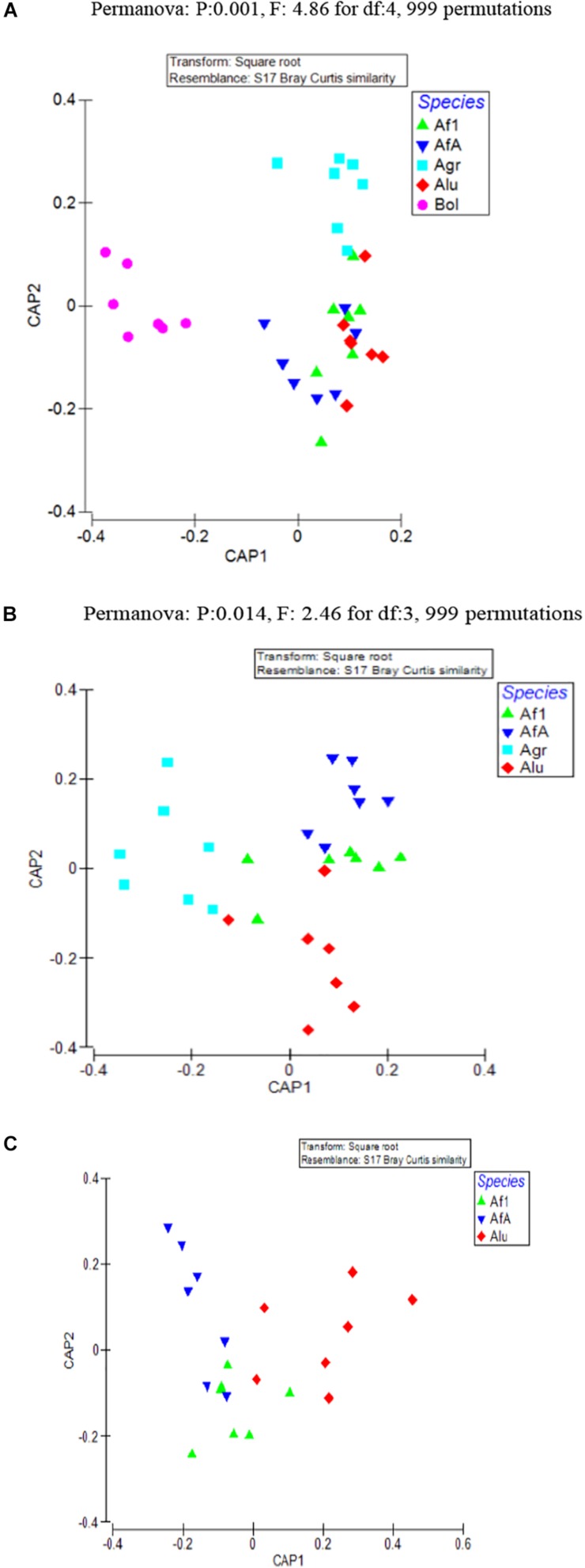
CAP was used to find axes that best discriminate the groups of interest. In this analysis, clustering at insect species level was examined. **(A)** All samples representing the five laboratory populations are included **(B)** Only the four *Anastrepha* laboratory populations (*Anastrepha grandis, Anastrepha ludens*, and *Anastrepha fraterculus*) are included. **(C)** Only the three laboratory populations from the *A. fraterculus* intergeneric group (*A. ludens* and *A. fraterculus*) are included. Af1, *A. fraterculus* sp. 1; AfA, *A. fraterculus* (Andean lineage); Agr, *A. grandis*; Alu, *A. ludens*; Bol, *B. oleae.*

As evident from CAP analysis (Figure 1A) there was a clear clustering of olive fruit fly samples against all others [tr(Q_m′HQ_m): 2.41921 P: 0.0001] and, after the removal of olive fruit fly from the analysis, *A. grandis* samples clustered together, although not very well separated from the remaining *Anastrepha* samples [tr(Q_m′HQ_m): 1.89387 P: 0.0001] (Figure 1B). After the removal of the Agr samples from the analysis, the remaining three *Anastrepha* colonies highly overlapped, although a tendency of the Alu samples to form a different cluster was observed (Figure 1C). When all populations were tested together, the Permanova analysis performed pointed to the statistically significant contribution of host species in the observed clustering (*p* = 0.001, *F* = 4.86, df: 4), which remained statistically significant after the removal of the Bol samples species (*p* = 0.014, *F* = 2.46, df: 3).

#### Developmental Stage and Age

**FIGURE 2:**
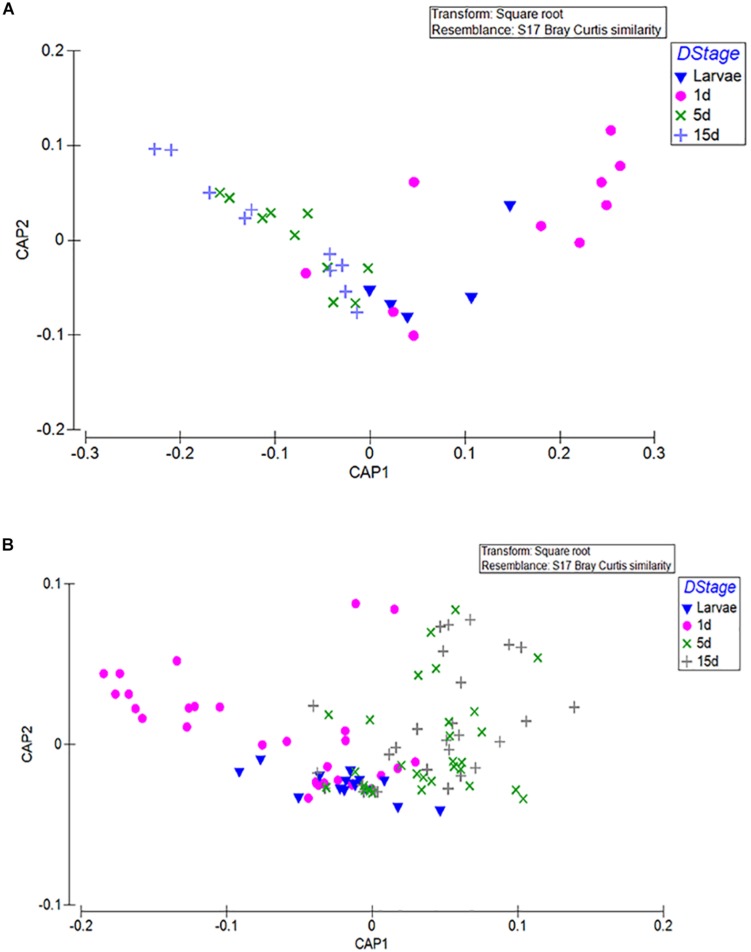
CAP was used to find axes that best discriminate the groups of interest. In this analysis, clustering based on developmental stage and age was tested. **(A)** The mean values of the 3 replicates for the male and female samples are displayed. **(B)** All replicates are displayed. L, larvae; M, males; F, females; 1D, 1-day old, unfed; 5D, 5–10-days old; 15D, 15–20-days old.

An obvious clustering in almost all laboratory populations was that of larvae and 1-day old flies (unfed) against older fed flies (Permanova; *p* < 0.001 1d/Larvae, Permanova; *p* < 0.001 1-day/10-day, Permanova; *p* < 0.001 1-day/15-day). A second level of clustering was that of the bacterial profile of the 5–10-days with the 15–20-days old flies (Permanova; *p* < 0.483). This was clear in Af1 (Supplementary Figure S3), AfA (Supplementary Figure S5), and Agr (Supplementary Figure S9) but not so in Alu (Supplementary Figure S7) and Bol (Supplementary Figure S11). CAP analysis of the different groups based on developmental stage and age indeed verified the separation of larvae and 1-day old adults from the 5 to 10 and 15 to 20 days-old adults [tr(Q_m′HQ_m): 0.7546 P: 0.0057] (Figure 2).

#### Fly Sex

**FIGURE 3:**
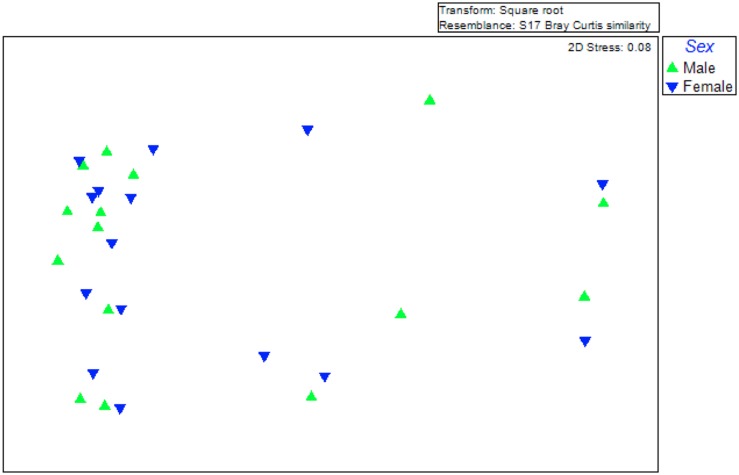
Multidimensional scaling (MDS) plot for a graphical illustration of changes in the bacterial community structure. Green triangles represent males while inverted blue triangles represent females. Similarities in ribotype profiles were calculated by the Brey-Curtis algorithm.

Another factor that can contribute to differences among symbiotic communities is the fly sex. Our data were not in favour of this hypothesis. An MDS analysis was performed, assuming two different groups: males and females. As evident in Figure 3, there was no obvious clustering based on the sex and the Permanova analysis gave no statistical support (Permanova; *p* < 0.984). Therefore, at least for the laboratory populations studied, sex cannot be considered as a factor contributing to the differences observed among the gut symbiotic communities.

### Key Players Constituting the Gut Symbiotic Communities of the Laboratory Populations

**FIGURE 4:**
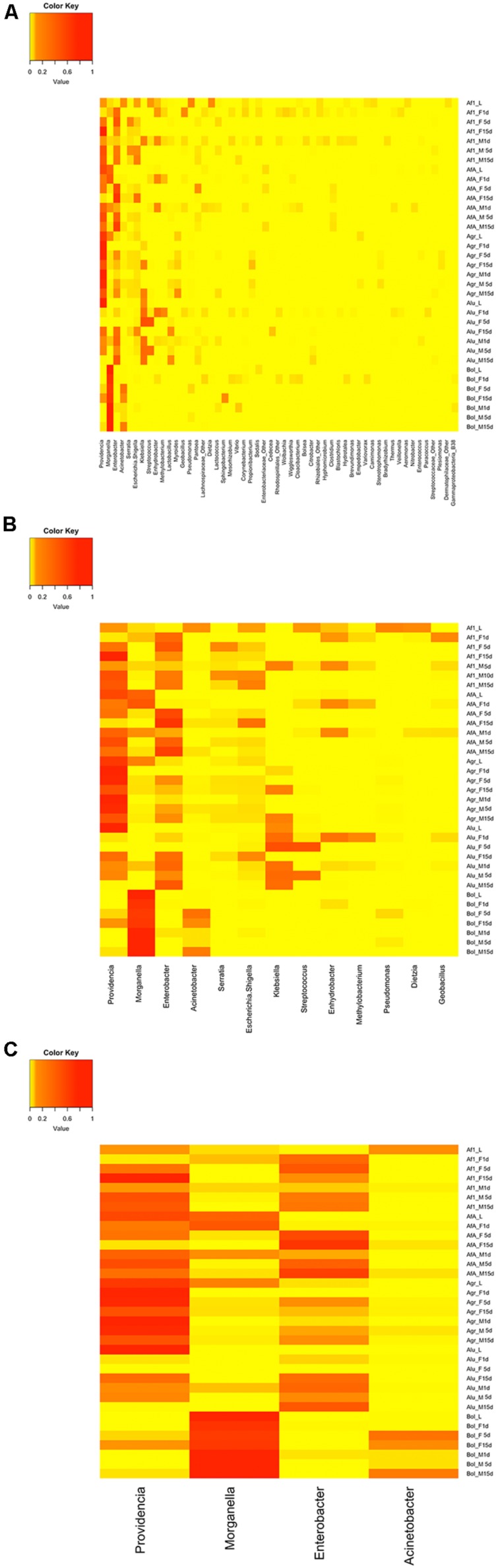
Heat maps showing the RA of the different OTUs, at genus level. **(A)** OTUs with a RA >1% **(B)** the thirteen most relative abundant OTUs **(C)** the four most relative abundant OTUs. Af1, *A. fraterculus* sp. 1; AfA, *A. fraterculus* (Andean lineage); Agr, *A. grandis*; Alu, *A. ludens*; Bol, *B. oleae*; L, larvae; M, males; F, females; 1D, 1-day old, unfed; 5D, 5–10-days old; 15D, 15–20-days old; G, gut.

Although five different laboratory populations were tested, representing five different species from two different genera, with samples across life cycle, only a limited number of bacterial OTUs displayed high RAs, as shown in the heat map of the OTUs (Figures 4A–C). Despite the presence of more than 400 different OTUs, only 53 had a RA of >1% in the different samples (Figure 4A). The 13 most abundant OTUs accounted for the 90% of the overall sequences detected, ranging from 64 to 100% per sample (Figure 4B). Unambiguously, the most abundant OTUs are *Providencia, Enterobacter*, and *Morganella* (Figure 4C), which account for the 32%, 20%, and 16% of the total sequences, respectively. However, *Morganella’s* abundance is mainly restricted to all olive fruit fly samples, where it can reach up to 100%, while *Providencia* and *Enterobacter* have a more “universal” distribution, present in high RAs in all species analyzed.

### Did We Miss Somebody? the Mock Experiment of “Expected” Tenants

Available sequences representing 28 different bacterial genera previously reported to be present in the gut symbiotic communities of different Tephritidae were downloaded and incorporated in our data set. QIIME was used to assign them to respective OTUs (Supplementary Table S2). In most of the taxa, all sequences were assigned to the correct OTU (Supplementary Figures S16A,B).

## Discussion

The present analysis indicates that the laboratory populations of the five targeted taxa harbored a varying degree of gut bacterial community diversity. Although the major “players” belonged to Enterobacteriaceae and the overall diversity can be considered rather low, compared to available data from natural populations of some of the species, there were clear differences among our samples. The structure of the communities can be considered as “dynamic,” since there were clear intra-population differences, based on the developmental stage and age. Moreover, there were clear inter-population differences, which can be attributed to a variety of factors, such as the original microbiome of the wild populations at the moment of colonization, the degree of adaptation and rearing conditions, but not sex.

### Key Players Constituting the Gut Symbiotic Communities of Tephritids

The data presented above are in line with previous studies in laboratory and natural populations of different tephritids. Up to now, the tephritid species model for such studies is the medfly, followed by the olive fruit fly and other *Bactrocera* spp. Previous studies have addressed several questions regarding laboratory and natural populations of the Mediterranean fruit fly. Marchini et al. (2002), using culture dependent approaches and classical microbiological techniques, suggested that *K. oxytoca* is dominating natural populations while *Enterobacter* sp. is dominating laboratory populations of the species. Several studies from another research team (Behar et al., 2005, 2008; Ben-Yosef et al., 2008; Ben Ami et al., 2010; Gavriel et al., 2011; Aharon et al., 2012) provided interesting findings that can be summarized as: (a) Enterobacteriaceae was shown to be the dominant community in the medfly gut, with relatively few genera being present in varying RAs, (b) *Klebsiella* is believed to be a key genus, important for the fitness of medfly and is mainly found in the wild populations of the species, (c) gut symbiotic community is dynamic, depending mainly on the developmental stage and age of adults and, (d) wild populations seem to harbor more polymorphic symbiotic communities than long adapted laboratory strains, although no direct comparison has been performed. More recently, it has been shown that plant host and instar stage are among the factors that shape gut symbiotic communities of natural populations (Malacrino et al., 2018). The reduced diversity of the gut symbiotic communities of laboratory populations of the Mediterranean fruit fly has been addressed by other groups, especially for the VIENNA 7 and VIENNA 8 genetic sexing strains (GSS) (Hamden et al., 2013; Augustinos et al., 2015). As an extreme example of reduced symbiotic diversity, Morrow et al. (2015) managed to retrieve only *Enterobacter* sp. from the VIENNA 7 GSS (a line reared in a mass rearing facility in Australia), even though high throughput 454 NGS sequencing was used.

In the olive fruit fly, all published data suggest that the diversity of the symbiotic community of this species is relatively low, with *Candidatus* Erwinia dacicola dominating wild populations, although other bacterial species, such as *Providencia* sp., *Enterobacter* sp. and *Acetobacter tropicalis* can be detected (Kounatidis et al., 2008; Sacchetti et al., 2008, 2014; Crotti et al., 2010; Estes et al., 2012a; Ben-Yosef et al., 2014; Koskinioti et al., 2019). At the same time, some of these studies presented important findings for the laboratory adaptation of the species, including the loss of *Candidatus* E. dacicola when olive fruit fly is reared on a totally artificial diet (Kounatidis et al., 2009; Estes et al., 2012a) and the increase of *Morganella* sp. in some of the olive fruit fly laboratory populations, which may be pathogenic and is considered as a negative symptom for artificially rearing (Estes et al., 2012b). Our data agree with what is expected for domesticated populations reared on totally artificial diet, since we did not retrieve *Candidatus* Erwinia dacicola sequences and *Morganella* sp. was dominating all samples of our olive fruit fly laboratory population (Supplementary Figure S10).

Studies regarding gut symbiotic communities in *Anastrepha* species are limited. The analysis of new and old laboratory populations of *A. ludens*, using culture dependent approaches, gave a total of 18 bacterial species belonging mainly to Enterobacteriaceae, with *Enterobacter*, *Providencia*, *Serratia*, and *Staphylococcus* being the most abundant genera (Kuzina et al., 2001). More recently a study in four *Anastrepha* species, namely *A. ludens, A. obliqua, A. serpetina*, and *A. striata*, using 454 pyrosequencing and samples collected from the nature, provided further insight in the symbiotic communities of this genus (Ventura et al., 2018). Four phyla were identified, with *Proteobacteria* being the dominant phylum. A total of 27 bacterial genera were identified, with *Citrobacter*, *Enterobacter*, *Escherichia*, *Klebsiella*, and *Raoultella*, being the most abundant. Our data are in line with previous studies, at least regarding the “key” players, which seem to be mainly few genera belonging mainly to Gammaproteobacteria.

Besides these, the structure of the gut symbiotic communities has been addressed in few other *Bactrocera* species (Wang et al., 2011; Prabhakar et al., 2013; Khan et al., 2014; Pramanik et al., 2014; Reddy et al., 2014; Wang A. et al., 2014; Wang H. et al., 2014; Andongma et al., 2015; Deutscher et al., 2017, 2018), suggesting that few genera are present in their gut symbiotic communities.

### Factors Shaping the Symbiotic Profile of Domesticated Populations

If phylogenetic distances of the species are responsible (to a certain extent) for the observed differences, one major clustering should be the one of *Anastrephas* against samples from the olive fruit fly, since they belong to different tephritid genera. Moreover, they have a completely different geographic distribution (*Anastrepha* species derive from Latin America, while the olive fruit fly derives from Europe). On a second level, after the removal of olive fruit fly from the analysis, *A. grandis* should cluster apart from the remaining *Anastrephas*, since this species belongs to a different intrageneric group, while both *A. fraterculus* and *A. ludens* belong to the *A. fraterculus* intrageneric group. Although our data are in line with this scenario, interpretation of these results is not easy and straightforward, since different factors highly overlap with each other (for example, phylogenetic differences overlap with different rearing practices). Olive fruit fly that had the most divergent symbiotic community is indeed the most distant phylogenetically and its rearing protocol is again different from all others. Finally, we must keep in mind that olive fruit fly is the only one of these species that is considered as strictly monophagous at the larval stage, while all others are polyphagous (with *A. grandis* having preference for cucurbitaceous fruits), which may influence the “build-up” of preferential symbiotic relationships in nature and through this, the original symbiotic “load” that was transferred in the laboratory and the potentially any novel symbiotic relationships established thereafter. The same applies for the remaining samples after the removal of the olive fruit fly from the analysis. *A. grandis*, which is phylogenetically distant from the other three *Anastrepha* colonies, also had a differentiated symbiotic community profile. However, larval stage diet of this species is still semi-artificial, differing from the other *Anastrepha* populations. Finally, the other three laboratory populations share phylogenetic proximity, long established adaptation, and common rearing practices.

Our analysis showed that developmental stage and age are important factors shaping symbiotic communities. Larvae and 1-day old adults have a different profile from older flies (5–10 and 15–20 days old). This has been previously shown in other studies dealing with the changes of symbiotic communities during development and/or age in the medfly (Ben Ami et al., 2010; Hamden et al., 2013; Augustinos et al., 2015; Malacrino et al., 2018). On the other hand, our data do not support a possible effect of the sex on gut symbionts, and there is still lack of studies addressing this factor in tephritids.

### Laboratory vs. Natural Populations – Who to Trust?

Laboratory populations have certain limitations regarding the deduction of generalized conclusions. High selective pressure, bottleneck effects, genetic drift and inbreeding are known to affect the genetic structure of population in the laboratory, mainly through reducing diversity. Although not tested directly yet, this could be the case for the symbiotic communities as well. Moreover, the limited “resources” for acquiring bacteria and the specific rearing practices (both in larvae and adult diet but also in oviposition) can lead to a laboratory symbiotic community much different than the one of the wild populations. Preferential adaptation of specific members of the symbiotic community, abundance of specific taxa in the restricted diet and redundancy of previously important symbionts in these new, stringent conditions could also lead to significant changes in the symbiotic status. An extreme example, also pointed out in this study (and previous ones), is the relationship of the olive fruit fly with *Candidatus* E. dacicola. All studies up to now suggest that this bacterium is necessary in natural populations or laboratory populations reared on olive fruits but becomes “unnecessary” in a totally artificial diet and gradually disappears (Capuzzo, 2005; Kounatidis et al., 2009; Estes et al., 2012a; Ben-Yosef et al., 2014; Koskinioti et al., 2019).

On the other hand, data collected from flies in nature could be considered as a “snapshot” of the symbiotic community, and we cannot be sure whether they correctly represent the “core” symbiotic needs of the different species. The random acquisition of bacteria from the different hosts, even if they are not essential, can also compromise the final conclusions. Colonized material enables organizing exact collections schemes, generating the replicates necessary for robust conclusions and allows “revisiting” in the future. Unfortunately, such experiments cannot be easily performed on natural populations. A combination of different approaches, starting from well characterized “wild” material and follow up for many generations in the laboratory will provide a more complete picture of the changes occurring in the symbiotic communities during laboratory domestication.

### Comparing Apples and Oranges: How to Correlate With Previous Findings?

As discussed above, there are several studies addressing the structure of gut symbiotic communities in different tephritids. However, a direct comparison is very difficult to be done, since there are many methodological differences among them, as explained in the Introduction. Apart from those, there are other factors that make comparison even more difficult. The first is the material being used: there are studies using material derived from the wild, others are using populations colonized in semi-artificial conditions and others, like ours, are using laboratory populations adapted in totally and semi-artificial rearing. A second factor that should be considered is the samples used for these studies. Samples can be larvae, pupae, emerging flies or flies of specific age and specific sex. In cases where adult flies were collected directly from the field, age could not be accurately specified. Our data are in line and, to some extent, directly verify some of the previous reported findings for laboratory populations. More specifically, our analysis shows that: (a) members of the *Enterobacteriaceae* are dominating the gut symbiotic communities of the studied colonies, (b) only few key players are present, (c) although of relatively low diversity, gut symbiotic communities are dynamic, since we observed clear changes in the RA of the different bacterial genera, evident mainly after young flies start to feed and, (d) the profile of these communities and the profile of their change through time can be influenced by a variety of parameters, such as insect host (species, its geographic origin, host plant, etc.) and rearing practices (both diet and oviposition substrates). However, other suspected parameters, such as sex were not found important in our study.

## Conclusion

The present study clearly indicates that insect species, including those which are under artificial or semi-artificial laboratory rearing conditions, can establish in their gut sophisticated symbiotic associations with diverse bacterial species. Most of the gut-associated bacterial species in all five-insect species studied were members of Enterobacteriaceae. The overall bacterial diversity observed in our samples was low when compared to the diversity observed in natural populations. Taxonomy, diet, and developmental stage were found to be key factors influencing the structure of the symbiotic communities. The role of rearing conditions, the degree of laboratory adaptation, and the original microbiome of the wild populations at the moment of colonization may also be critical. These potential factors deserve additional investigation to assess the potential improvement in a cost-effective manner the rearing efficiency and the biological quality of mass reared insect species, which may be the target of AW-IPM strategies with a SIT component.

## Data Availability

The datasets generated for this study can be found in NCBI, PRJNA525967.

## Author Contributions

AA designed and performed the experiments, analyzed the data, and drafted part of the manuscript. GT analyzed the data and drafted part of the manuscript. CC interpreted the data and critically revised the manuscript. AMA designed the experiments, interpreted the data, and critically revised the manuscript. KB conceived and designed the experiments, interpreted the data, and drafted part of the manuscript. All authors approved the final version of the manuscript and agreed to be accountable for all aspects of the work in ensuring that questions related to the accuracy or integrity of any part of the work are appropriately investigated and resolved.

## Conflict of Interest Statement

The authors declare that the research was conducted in the absence of any commercial or financial relationships that could be construed as a potential conflict of interest.

## Footnotes

1 https://dna.macrogen.com/eng/
