# Supplementary material for: Taxonomy, Diet, and Developmental Stage Contribute to the Structuring of Gut-Associated Bacterial Communities in Tephritid Pest Species
Source: Front Microbiol. 2019 Aug 29;10:2004. doi: 10.3389/fmicb.2019.02004 (PMC6727639; doi:10.3389/fmicb.2019.02004)
Supplement: Supplementary file 1 [file Table_1.DOCX]

Table S1: Summary of the gut collection scheme

| Species/Strain | Larvae | Males | | | Females | | | Total samples |
| --- | --- | --- | --- | --- | --- | --- | --- | --- |
|  | 3^rd^ instar | 1 day | 5-10 days | 15-20 days | 1 day | 5-10 days | 15-20 days |  |
| *A. fraterculus*  Argentina, Tucuman  (AfA) | 3x5 | 3x5 | 3x5 | 3x5 | 3x5 | 3x5 | 3x5 | 21 |
| *A. fraterculus*  ICA, Peru  Af1 | 3x5 | 3x5 | 3x5 | 3x5 | 3x5 | 3x5 | 3x5 | 21 |
| *A. grandis*  Brazil, Sao Paolo  Agr | 3x5 | 3x5 | 3x5 | 3x5 | 3x5 | 3x5 | 3x5 | 21 |
| *A. ludens*  Guatemala  Alu | 3x5 | 3x5 | 3x5 | 3x5 | 3x5 | 3x5 | 3x5 | 21 |
| *B. oleae*  Greece, Democritus lab  Bol | 3x5 | 3x5 | 3x5 | 3x5 | 3x5 | 3x5 | 3x5 | 21 |
| Total samples | 15 | 15 | 15 | 15 | 15 | 15 | 15 | 105 |

3x5: three replicas x 5 individual guts per replica
