# Supplementary material for: Taxonomy, Diet, and Developmental Stage Contribute to the Structuring of Gut-Associated Bacterial Communities in Tephritid Pest Species
Source: Front Microbiol. 2019 Aug 29;10:2004. doi: 10.3389/fmicb.2019.02004 (PMC6727639; doi:10.3389/fmicb.2019.02004)
Supplement: Supplementary file 4 [file Table_4.pdf]

Supplementary Material

Supplementary Figure 1: Rarefaction curves. Af1: *A. fraterculus* sp. 1; AfA: *A. fraterculus* (Andean lineage); Agr: *A. grandis*; Alu: *A. ludens*; Bol: *B. oleae* L: Larvae; M: Males; F: Females; 1D: 1-day old, unfed; 5D: 5-10-days old; 15D: 15-20-days old; G: Gut

Supplementary Figure 2: Diversity of the gut symbiotic community of the *A. fraterculus* sp.1 (Af1) laboratory population at **(A)** class level **(B)** family level **(C)** genus level.
Only OTUs with RA>0.01 in at least one sample are shown.

Supplementary Figure 3: PCoA analysis of the samples derived from the gut symbiotic communities of the *A. fraterculus* sp.1 (Af1) laboratory population.
(A) the three replications of each sample are shown as a single point **(B)** the three replications of each sample are shown.
M: male; F: female; L: 3rd instar larvae; 1d: 1-day old unfed adult; 5d: 5-10 days old adults; 15d: 15-20 days old adults.

Supplementary Figure 4: Diversity of the gut symbiotic community of the *A. fraterculus* (Andean lineage) (AfA) laboratory population at **(A)** class level **(B)** family level **(C)** genus level.
Only OTUs with RA>0.01 in at least one sample are shown.

Supplementary Figure 5: PCoA analysis of the samples derived from the gut symbiotic communities of the *A. fraterculus* (Andean lineage) AfA laboratory population.
(A) the three replications of each sample are shown as a single point **(B)** the three replications of each sample are shown.
M: male; F: female; L: 3rd instar larvae; 1d: 1-day old unfed adult; 5d: 5-10 days old adults; 15d: 15-20 days old adults.

Supplementary Figure 6: Diversity of the gut symbiotic community of the *A. ludens* (Alu) laboratory population at **(A)** class level **(B)** family level **(C)** genus level.
Only OTUs with RA>0.01 in at least one sample are shown.

Supplementary Figure 7: PCoA analysis of the samples derived from the gut symbiotic communities of the *A. ludens* (Alu) laboratory population.
(A) the three replications of each sample are shown as a single point **(B)** the three replications of each sample are shown.
M: male; F: female; L: 3rd instar larvae; 1d: 1-day old unfed adult; 5d: 5-10 days old adults; 15d: 15-20 days old adults.

Supplementary Figure 8: Diversity of the gut symbiotic community of the *A. grandis* (Agr) laboratory population at **(A)** class level **(B)** family level **(C)** genus level.
Only OTUs with RA>0.01 in at least one sample are shown.

Supplementary Figure 9: PCoA analysis of the samples derived from the gut symbiotic communities of the *A. grandis* (Agr) laboratory population.
(A) the three replications of each sample are shown as a single point **(B)** the three replications of each sample are shown.
M: male; F: female; L: 3rd instar larvae; 1d: 1-day old unfed adult; 5d: 5-10 days old adults; 15d: 15-20 days old adults.

Supplementary Figure 10: Diversity of the gut symbiotic community of the *B. oleae* (Bol) laboratory population at **(A)** class level **(B)** family level **(C)** genus level.
Only OTUs with RA>0.01 in at least one sample are shown.

Supplementary Figure 11: PCoA analysis of the samples derived from the gut symbiotic communities of the *B. oleae* (Bol) laboratory population.
(A) the three replications of each sample are shown as a single point **(B)** the three replications of each sample are shown.
M: male; F: female; L: 3rd instar larvae; 1d: 1-day old unfed adult; 5d: 5-10 days old adults; 15d: 15-20 days old adults.

Supplementary Figure 12: PCoA analysis of the samples derived from the gut symbiotic communities of the 3rd instar larvae of the five laboratory populations. the three replications of each sample are shown.

Supplementary Figure 13: PCoA analysis of the samples derived from the gut symbiotic communities of the 1-day old adults of the five laboratory populations.
(A) the three replications of each sample are shown as a single point **(B)** the three replications of each sample are shown.

Supplementary Figure 14: PCoA analysis of the samples derived from the gut symbiotic communities of the 5-10 days old adults of the five laboratory populations.
(A) the three replications of each sample are shown as a single point **(B)** the three replications of each sample are shown.

Supplementary Figure 15: PCoA analysis of the samples derived from the gut symbiotic communities of the 15-20 days old adults of the five laboratory populations.
(A) the three replications of each sample are shown as a single point **(B)** the three replications of each sample are shown.

Supplementary Figure 16: The mock experiment was performed using *16S rRNA* sequences of genera known to participate in gut symbiotic communities of Tephritidae. All known sequences of the 28 genera examined were retrieved from Greengenes and SILVA databases.
(A) Percentage of the retrieved sequences that were correctly assigned to the 28 genera examined by deploying the same QIIME pipeline with the one used for our dataset. Only sequences representing genera that have been underrepresented in our study were tested; **(B)** Percentage of the retrieved sequences that were correctly assigned to their respective genera, along with the percentage of sequences from the same genus present in the different colonies analysed in the present study.

Supplementary Table 1: Summary of the gut collection scheme.

Supplementary Table 2: List of bacterial taxa examined.

Supplementary Excel 1: Relative abundances at phylum, class, order, family, and genus level in all samples.

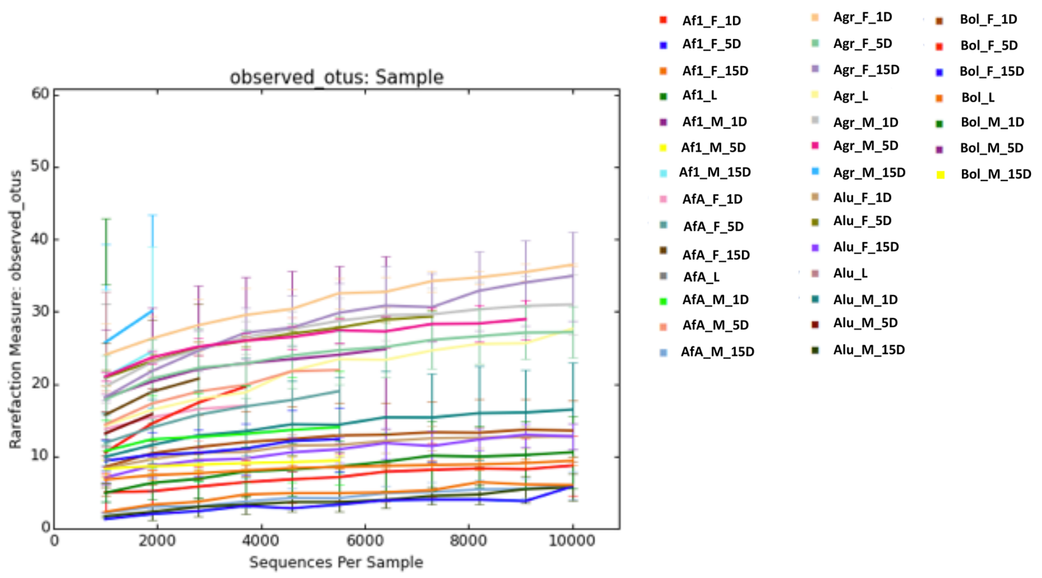

Figure S1

Supplementary Figure 1: Rarefaction curves. Af1: *A. fraterculus* sp. 1; AfA: *A. fraterculus* (Andean lineage); Agr: *A. grandis*; Alu: *A. ludens*; Bol: *B. oleae* L: Larvae; M: Males; F: Females; 1D: 1-day old, unfed; 5D: 5-10-days old; 15D: 15-20-days old; G: Gut

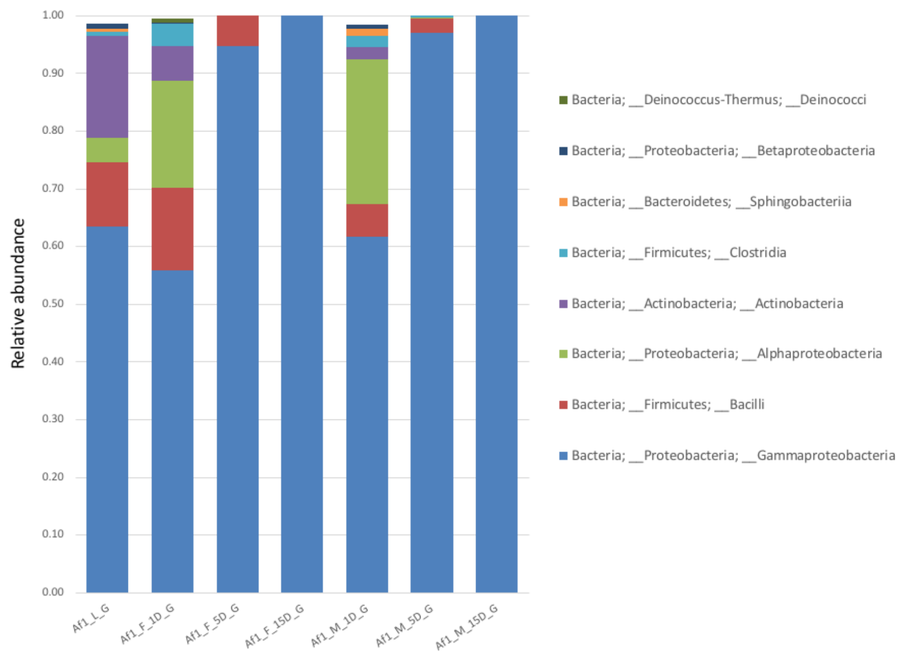

Figure S2A

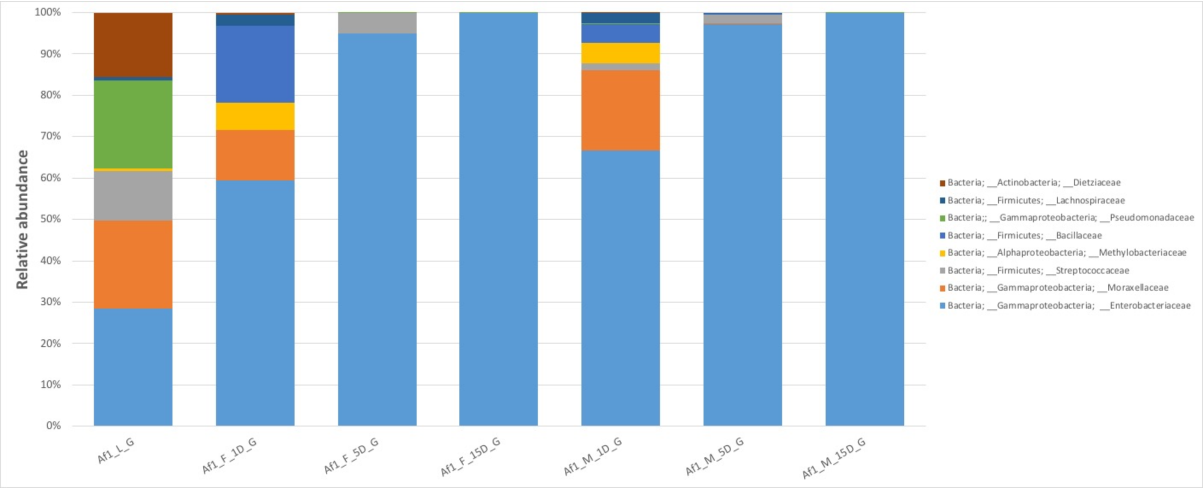

Figure S2B

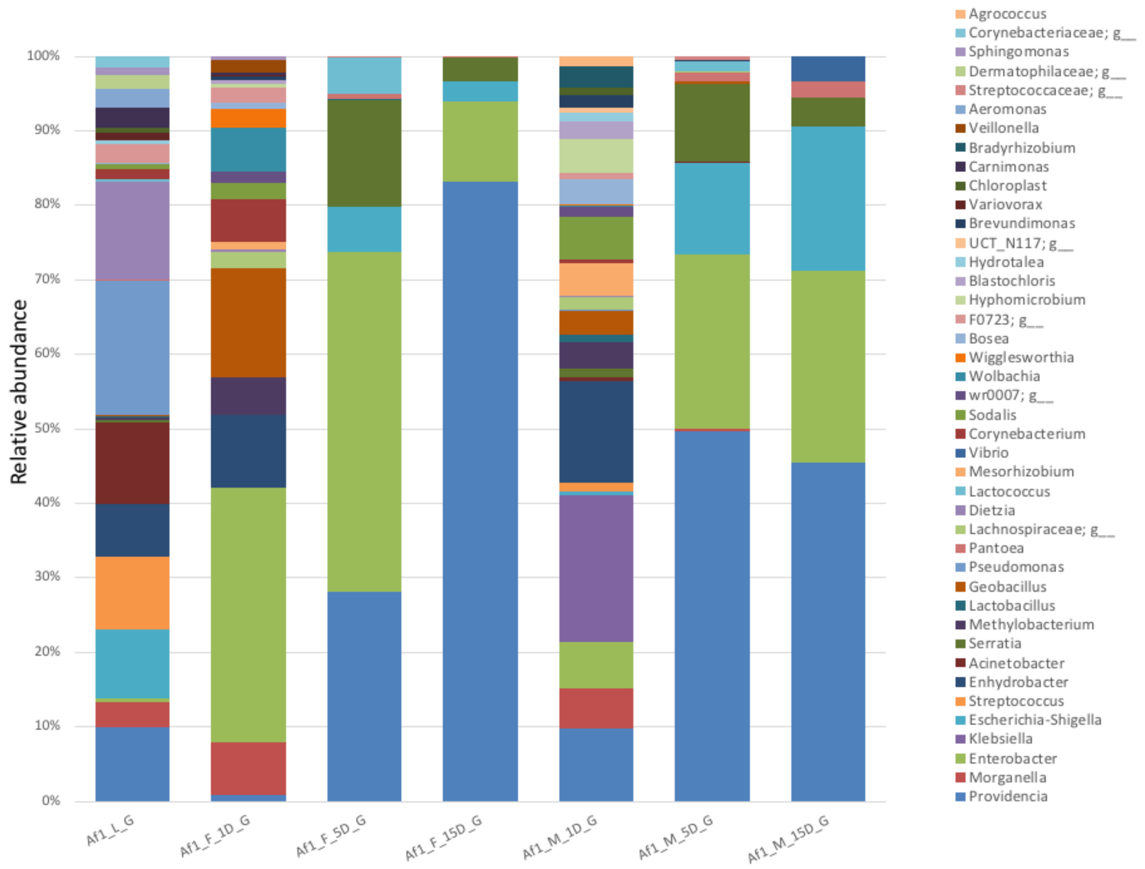

Figure S2C

Supplementary Figure 2: Diversity of the gut symbiotic community of the *A. fraterculus* sp.1 (Af1) laboratory population at **(A)** class level **(B)** family level **(C)** genus level.
Only OTUs with RA>0.01 in at least one sample are shown.

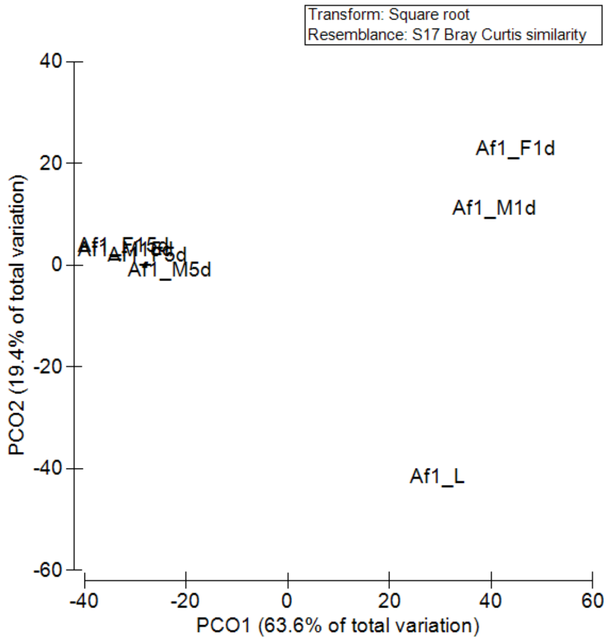

Figure S3A

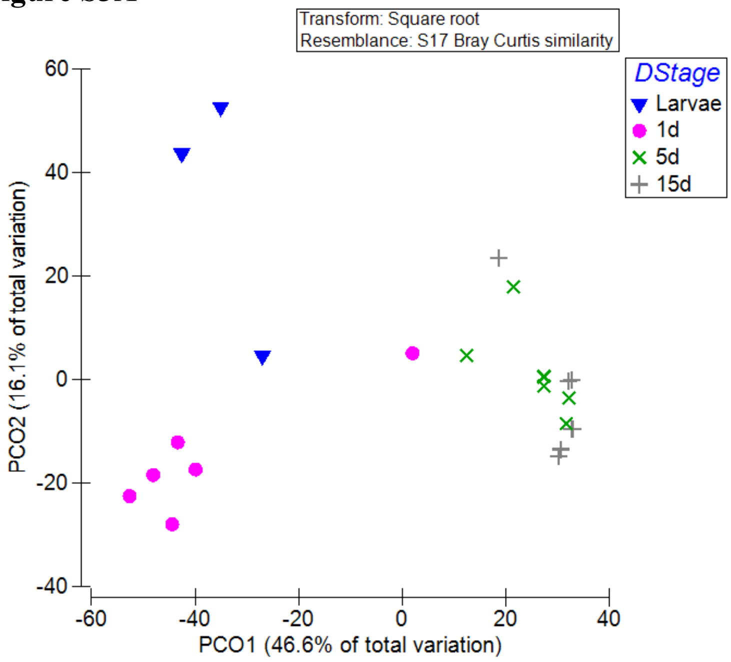

Figure S3B

Supplementary Figure 3: PCoA analysis of the samples derived from the gut symbiotic communities of the *A. fraterculus* sp.1 (Af1) laboratory population.

(A) the three replications of each sample are shown as a single point **(B)** the three replications of each sample are shown.

M: male; F: female; L: 3rd instar larvae; 1d: 1-day old unfed adult; 5d: 5-10 days old adults; 15d: 15-20 days old adults.

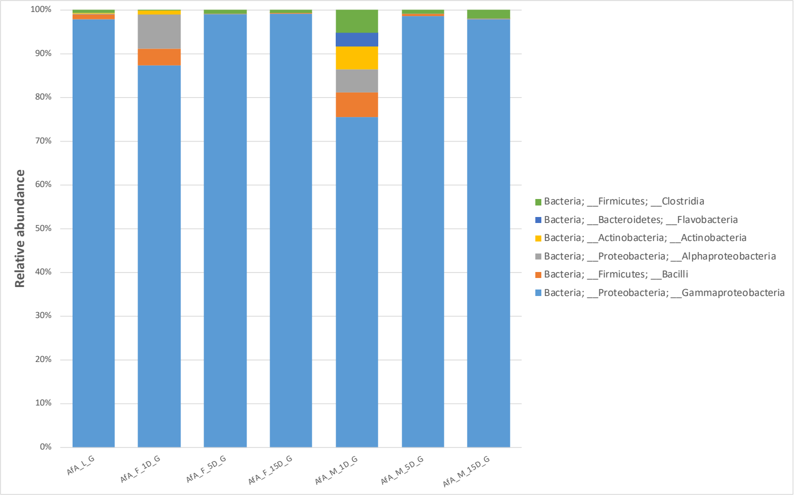

Figure S4A

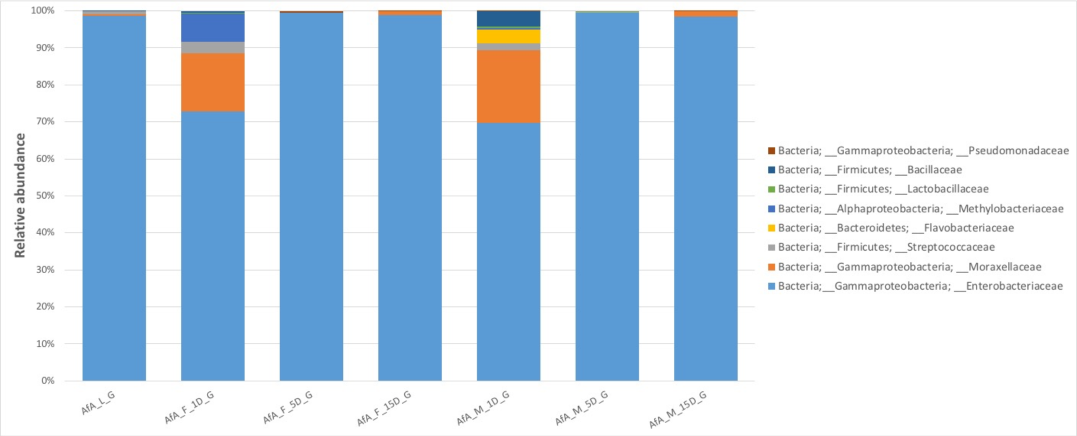

Figure S4B

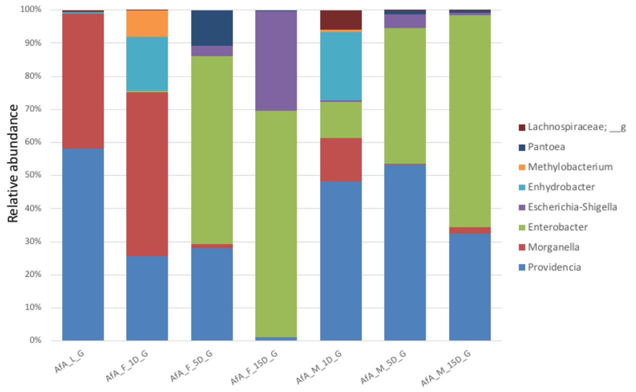

Figure S4C

Supplementary Figure 4: Diversity of the gut symbiotic community of the *A. fraterculus* (Andean lineage) (AfA) laboratory population at **(A)** class level **(B)** family level **(C)** genus level. Only OTUs with RA>0.01 in at least one sample are shown.

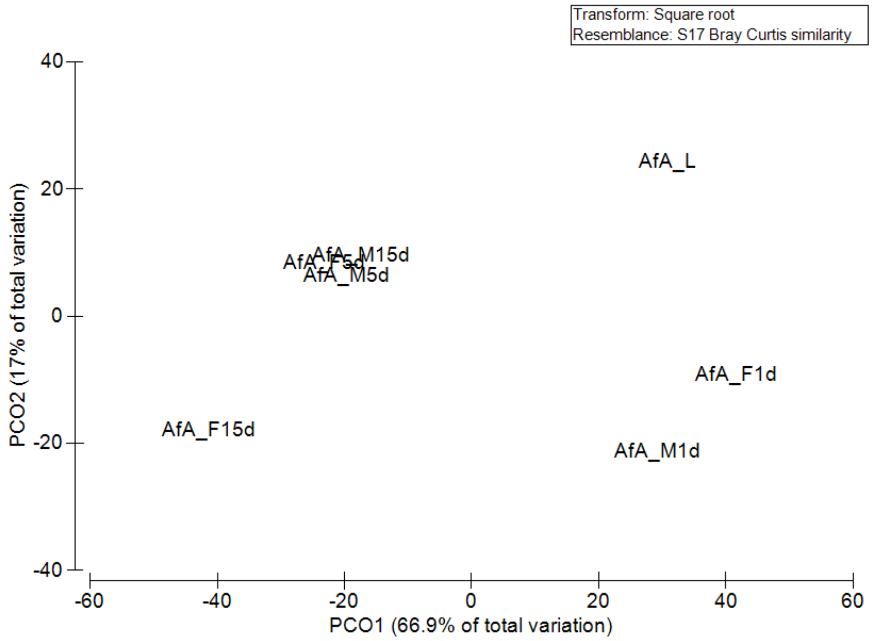

Figure S5A

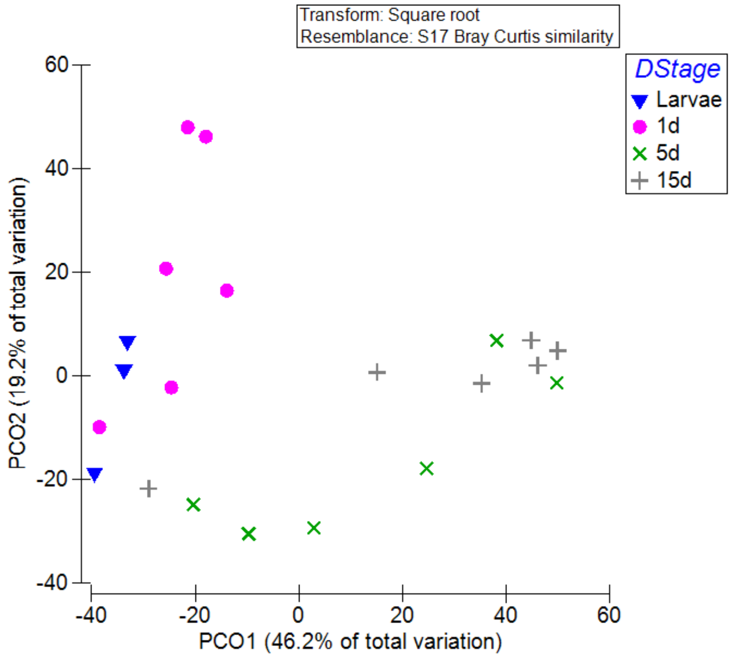

Figure S5B

Supplementary Figure 5: PCoA analysis of the samples derived from the gut symbiotic communities of the *A. fraterculus* (Andean lineage) AfA laboratory population.
(A) the three replications of each sample are shown as a single point **(B)** the three replications of each sample are shown.
M: male; F: female; L: 3rd instar larvae; 1d: 1-day old unfed adult; 5d: 5-10 days old adults; 15d: 15-20 days old adults.

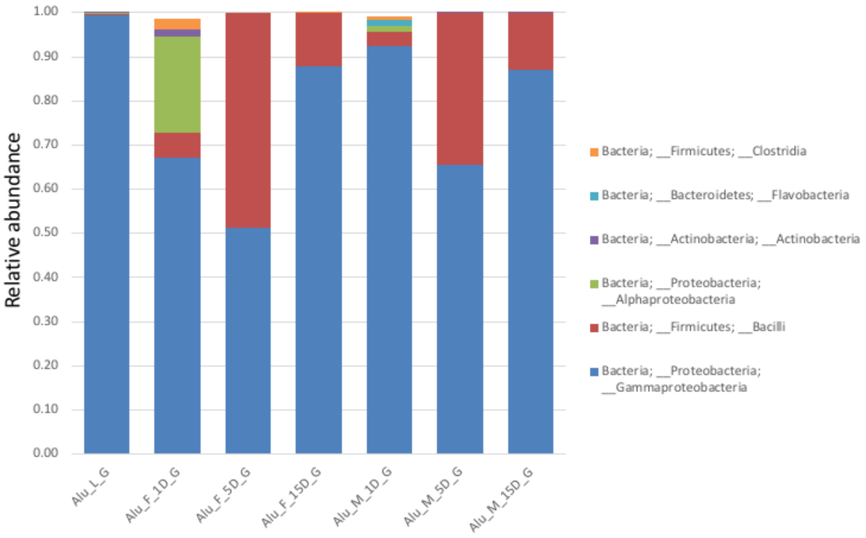

Figure S6A

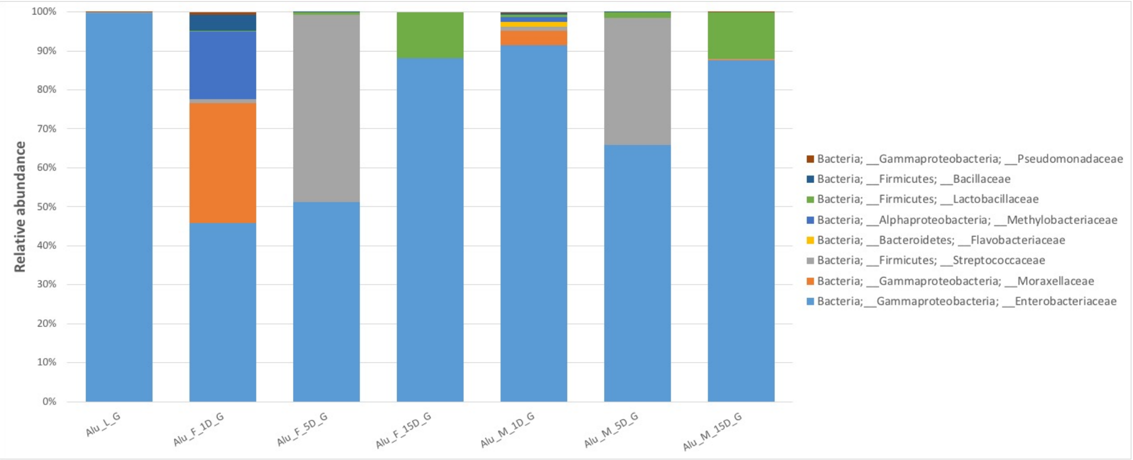

Figure S6B

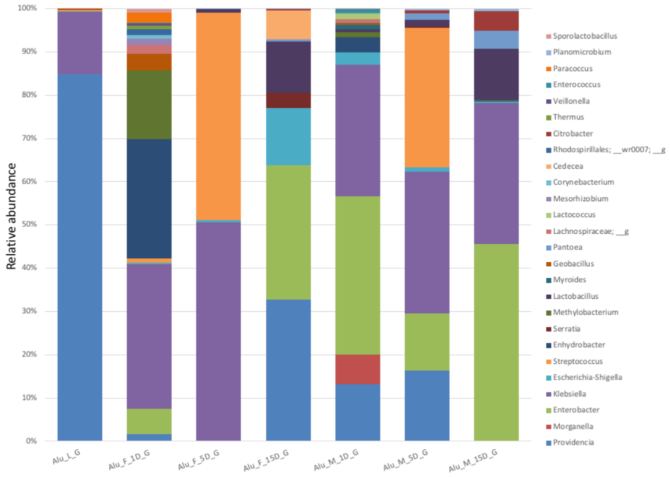

Figure S6C

Supplementary Figure 6: Diversity of the gut symbiotic community of the *A. ludens* (Alu) laboratory population at **(A)** class level **(B)** family level **(C)** genus level.
Only OTUs with RA>0.01 in at least one sample are shown.

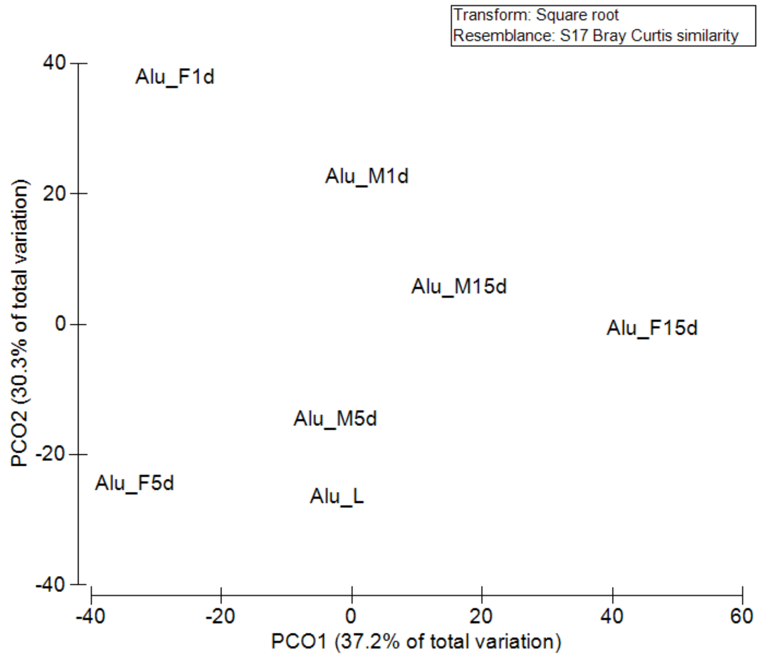

Figure S7A

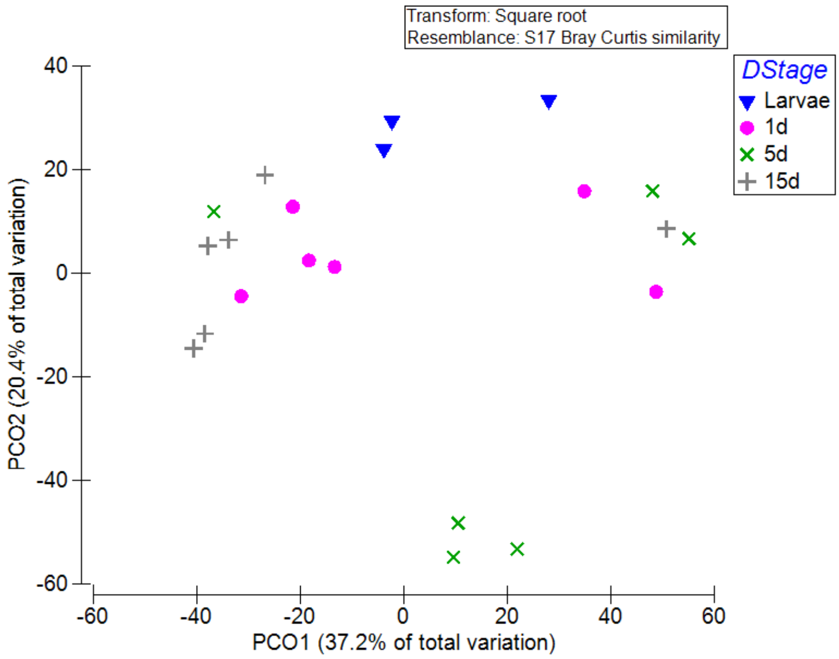

Figure S7B

Supplementary Figure 7: PCoA analysis of the samples derived from the gut symbiotic communities of the *A. ludens* (Alu) laboratory population.
(A) the three replications of each sample are shown as a single point **(B)** the three replications of each sample are shown.
M: male; F: female; L: 3rd instar larvae; 1d: 1-day old unfed adult; 5d: 5-10 days old adults; 15d: 15-20 days old adults.

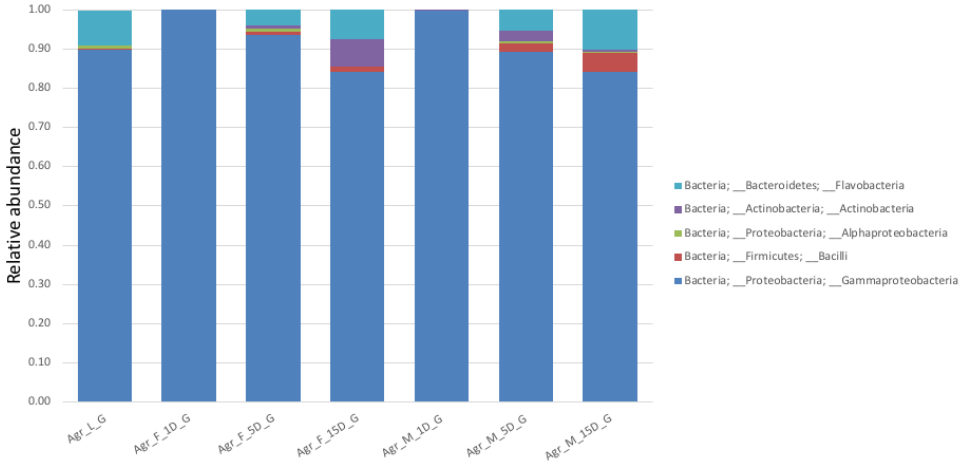

Figure S8A

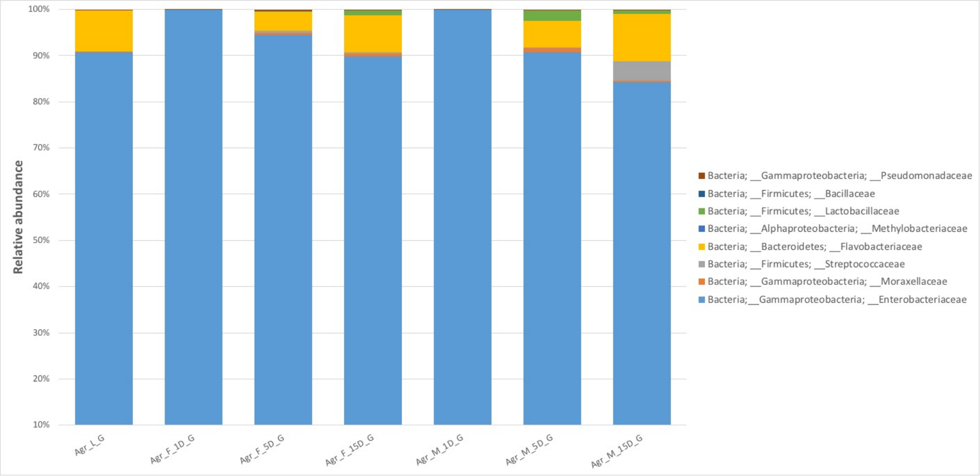

Figure S8B

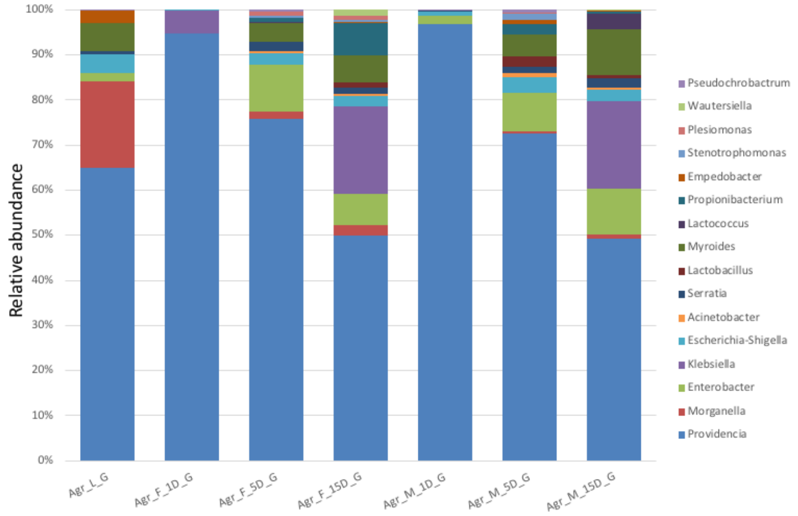

Figure S8C

Supplementary Figure 8: Diversity of the gut symbiotic community of the *A. grandis* (Agr) laboratory population at **(A)** class level **(B)** family level **(C)** genus level.
Only OTUs with RA>0.01 in at least one sample are shown.

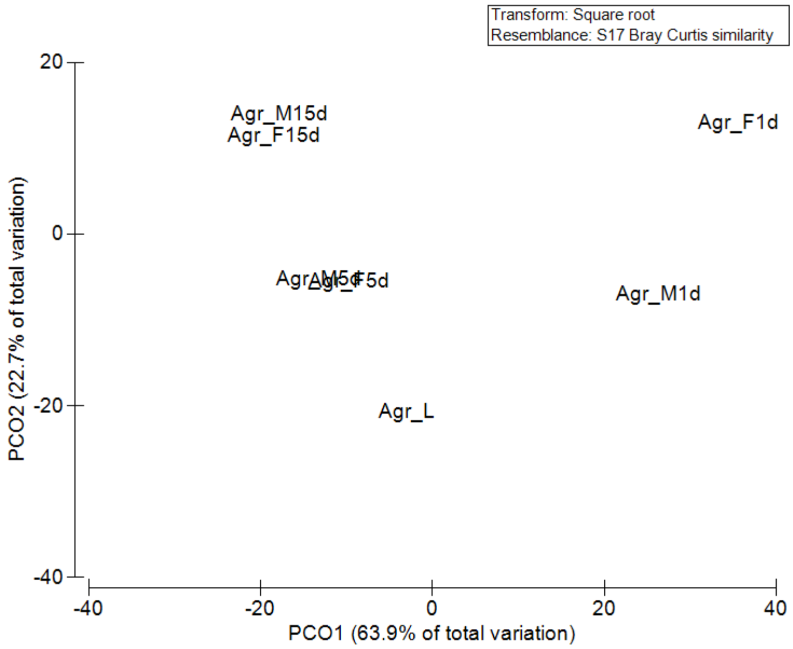

Figure S9A

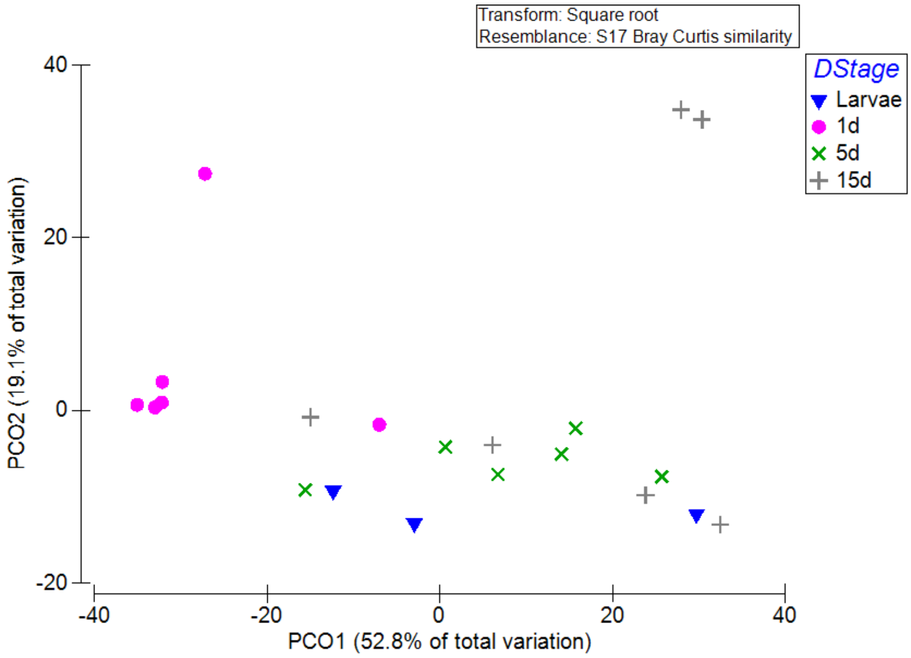

Figure S9B

Supplementary Figure 9: PCoA analysis of the samples derived from the gut symbiotic communities of the *A. grandis* (Agr) laboratory population.
(A) the three replications of each sample are shown as a single point **(B)** the three replications of each sample are shown.
M: male; F: female; L: 3rd instar larvae; 1d: 1-day old unfed adult; 5d: 5-10 days old adults; 15d: 15-20 days old adults.

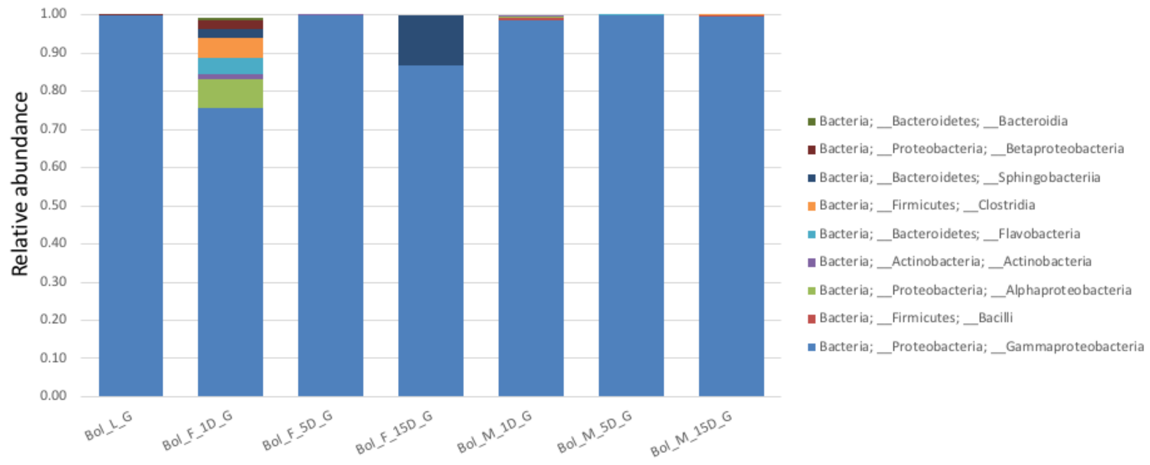

Figure S10A

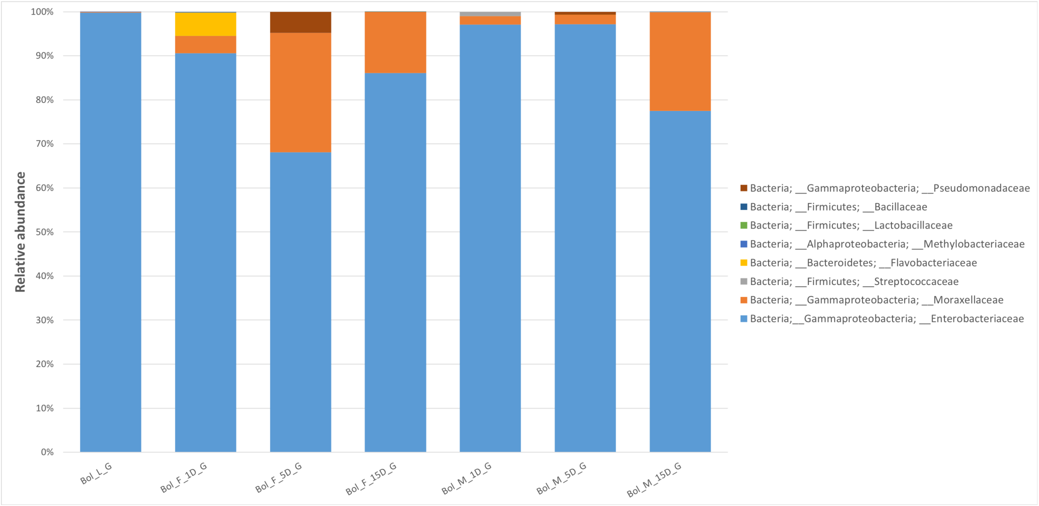

Figure S10B

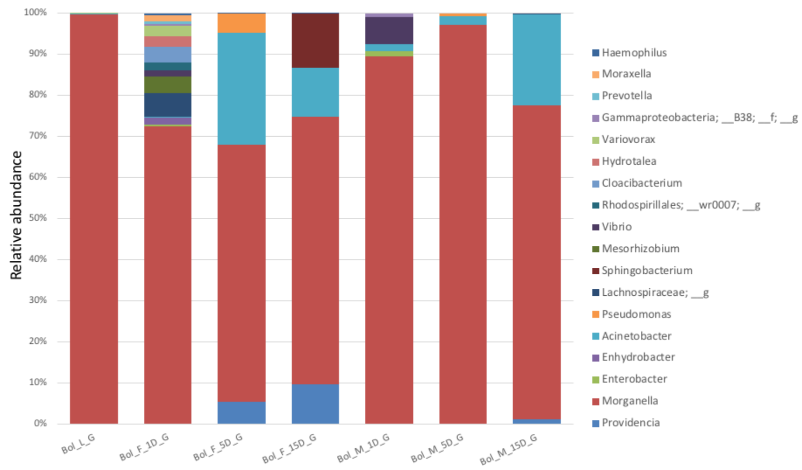

Figure S10C

Supplementary Figure 10: Diversity of the gut symbiotic community of the *B. oleae* (Bol) laboratory population at **(A)** class level **(B)** family level **(C)** genus level.
Only OTUs with RA>0.01 in at least one sample are shown.

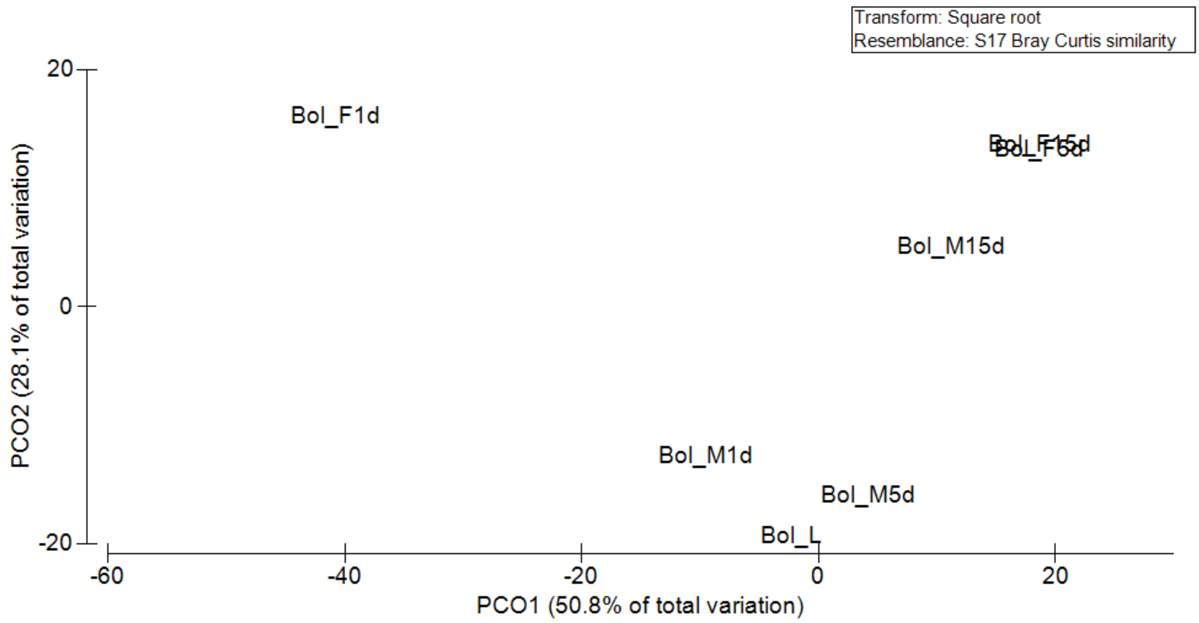

Figure S11A

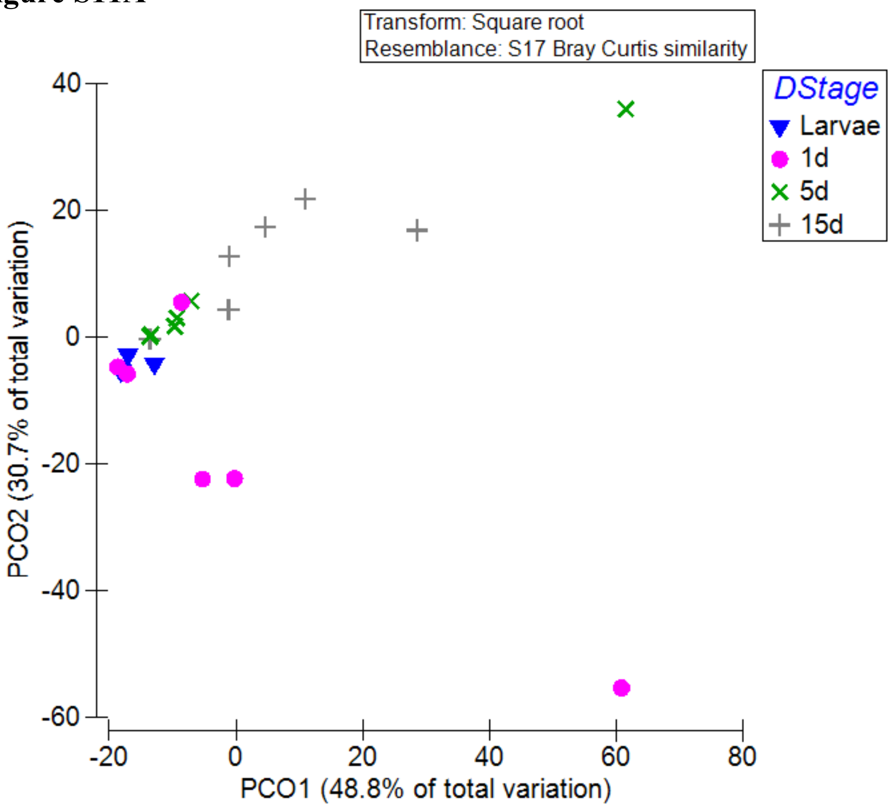

Figure S11B

Supplementary Figure 11: PCoA analysis of the samples derived from the gut symbiotic communities of the *B. oleae* (Bol) laboratory population.

(A) the three replications of each sample are shown as a single point **(B)** the three replications of each sample are shown.

M: male; F: female; L: 3rd instar larvae; 1d: 1-day old unfed adult; 5d: 5-10 days old adults; 15d: 15-20 days old adults.

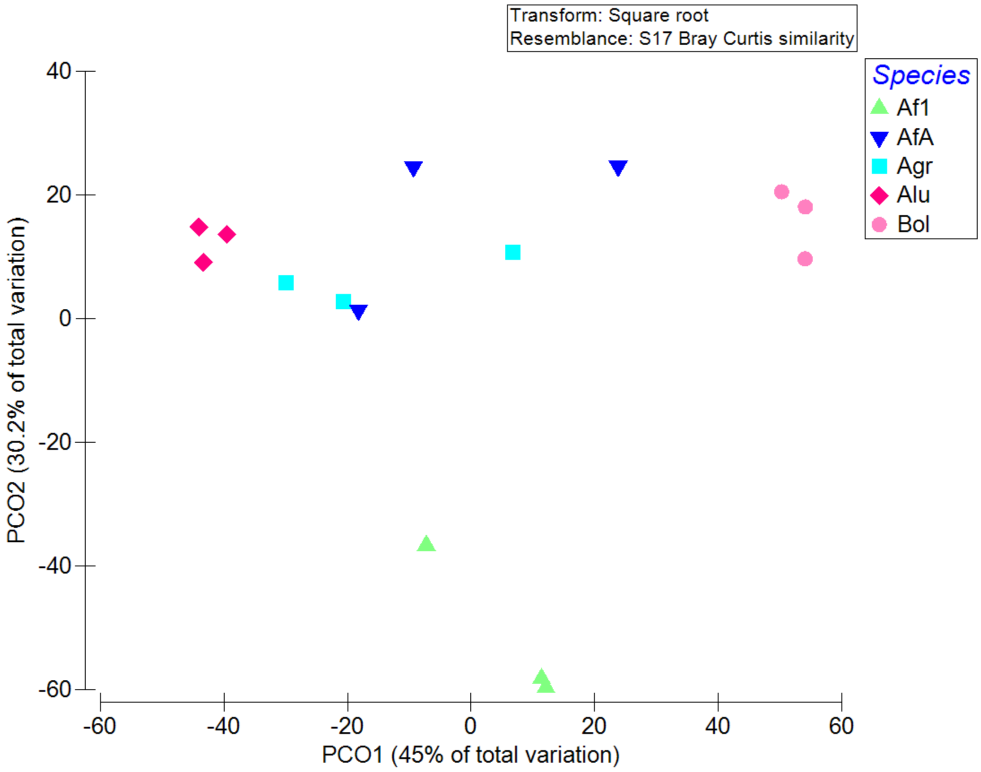

Figure S12

Supplementary Figure 12: PCoA analysis of the samples derived from the gut symbiotic communities of the 3rd instar larvae of the five laboratory populations. The three replications of each sample are shown. Af1: *A. fraterculus* sp. 1; AfA: *A. fraterculus* (Andean lineage); Agr: *A. grandis*; Alu: *A. ludens*; Bol: *B. oleae*

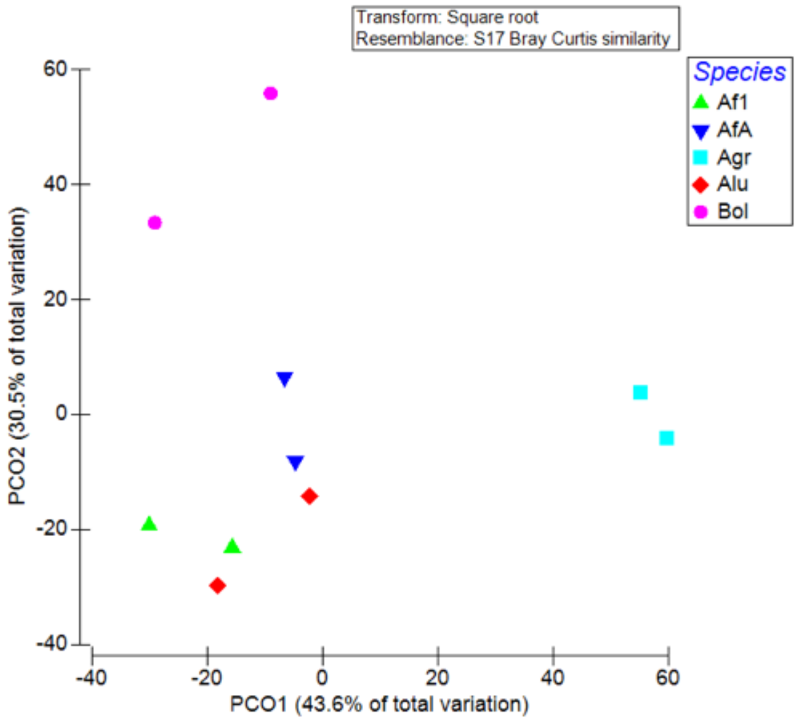

Figure S13A

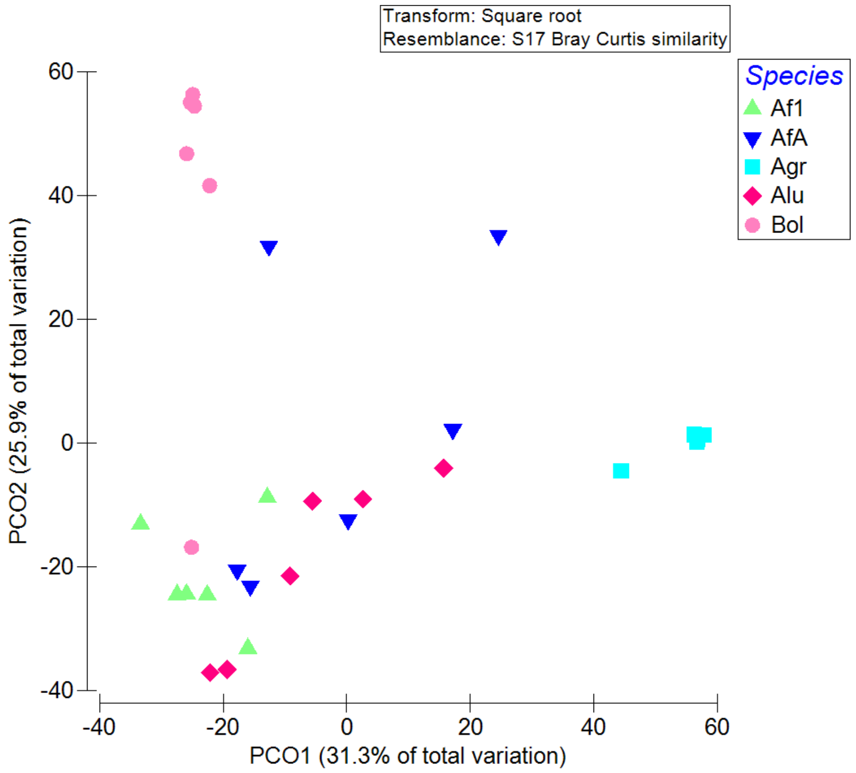

Figure S13B

Supplementary Figure 13: PCoA analysis of the samples derived from the gut symbiotic communities of the 1-day old adults of the five laboratory populations.

(A) the three replications of each sample are shown as a single point **(B)** the three replications of each sample are shown. Af1: *A. fraterculus* sp. 1; AfA: *A. fraterculus* (Andean lineage); Agr: *A. grandis*; Alu: *A. ludens*; Bol: *B. oleae*

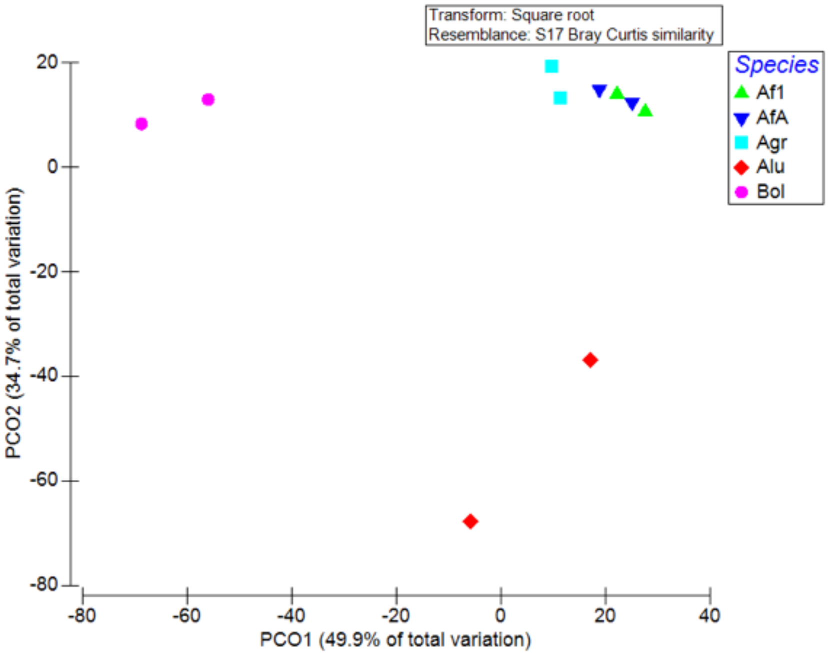

Figure S14A

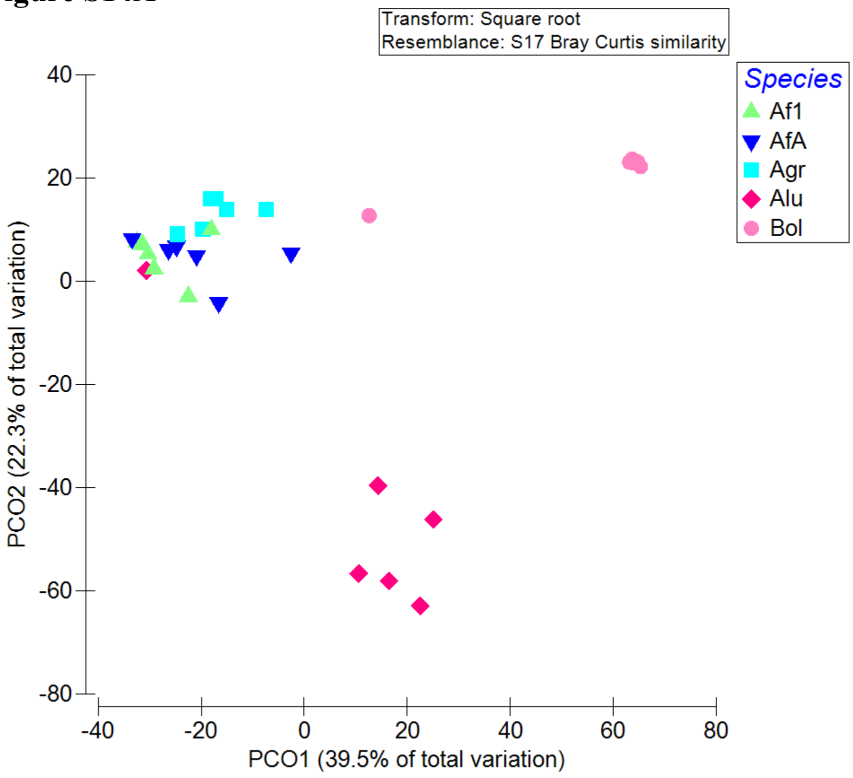

Figure S14B

Supplementary Figure 14: PCoA analysis of the samples derived from the gut symbiotic communities of the 5-10 days old adults of the five laboratory populations.
(A) the three replications of each sample are shown as a single point **(B)** the three replications of each sample are shown. Af1: *A. fraterculus* sp. 1; AfA: *A. fraterculus* (Andean lineage); Agr: *A. grandis*; Alu: *A. ludens*; Bol: *B. oleae*

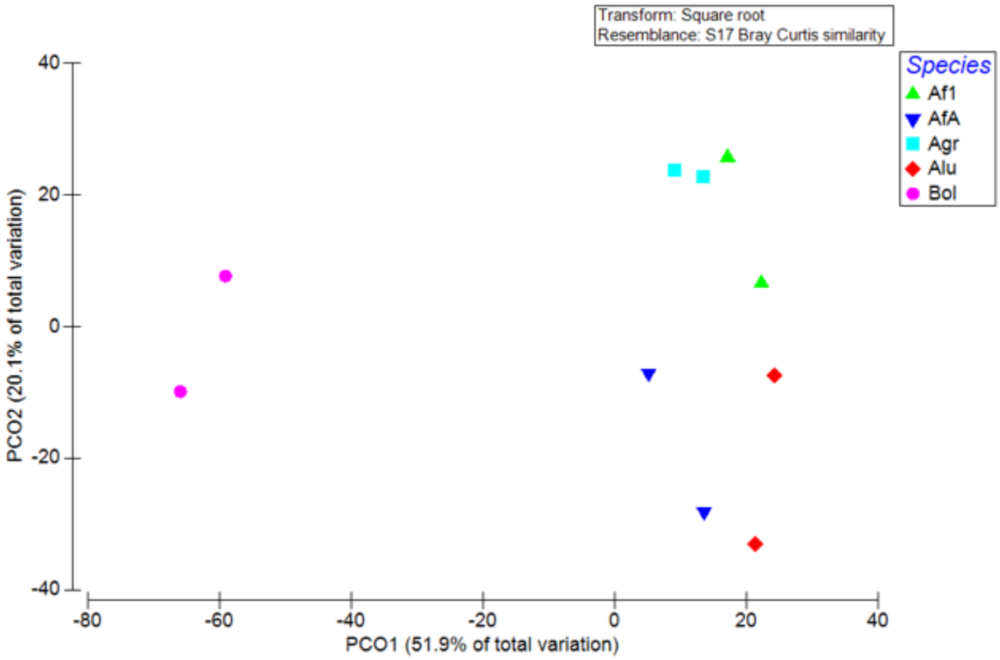

Figure S15A

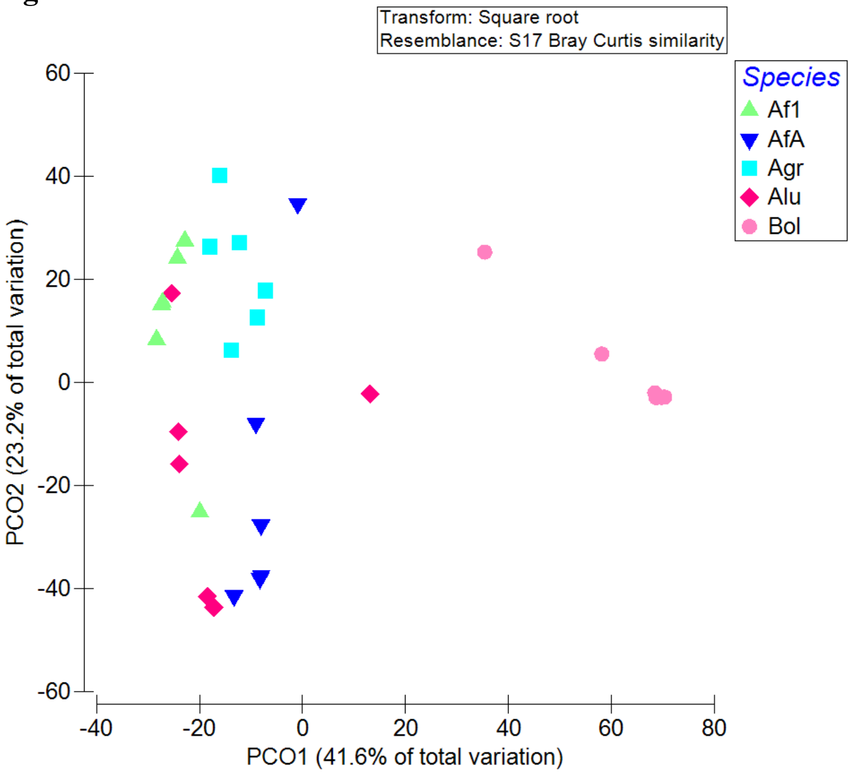

Figure S15B

Supplementary Figure 15: PCoA analysis of the samples derived from the gut symbiotic communities of the 15-20 days old adults of the five laboratory populations.
(A) the three replications of each sample are shown as a single point **(B)** the three replications of each sample are shown. Af1: *A. fraterculus* sp. 1; AfA: *A. fraterculus* (Andean lineage); Agr: *A. grandis*; Alu: *A. ludens*; Bol: *B. oleae*

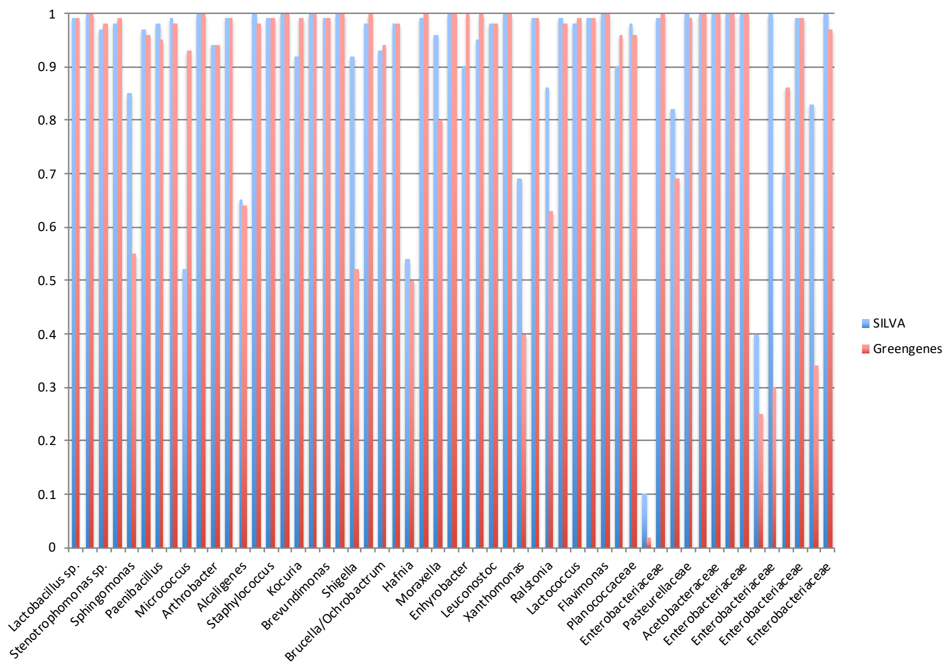

Figure S16A

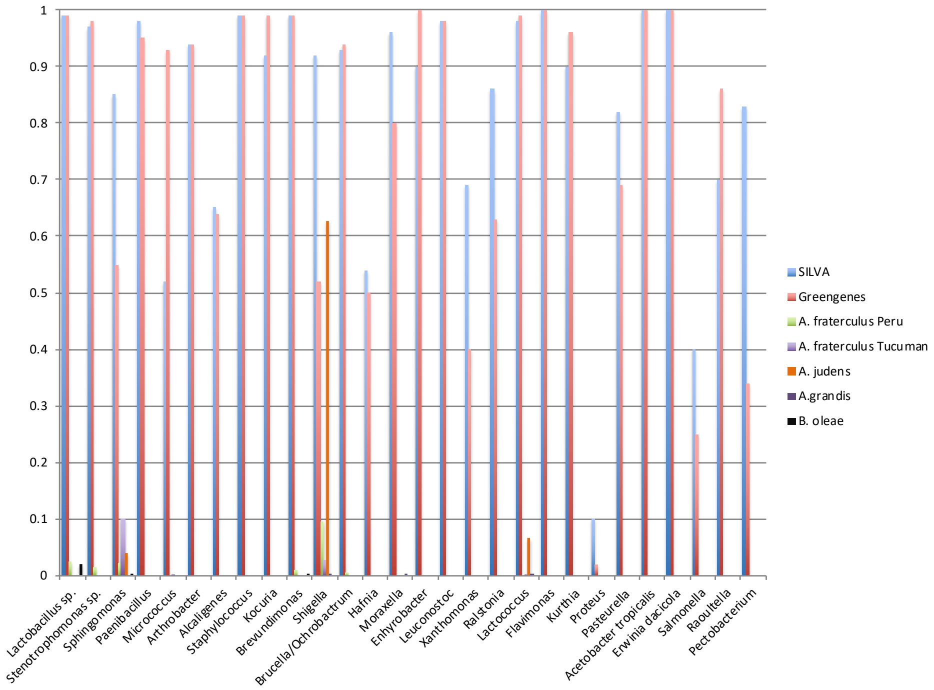

Figure S16B

Supplementary Figure 16: The mock experiment was performed using *16S rRNA* sequences of genera known to participate in gut symbiotic communities of Tephritidae. All known sequences of the 28 genera examined were retrieved from Greengenes and SILVA databases. **(A)** Percentage of the retrieved sequences that were correctly assigned to the 28 genera examined by deploying the same QIIME pipeline with the one used for our dataset. Only sequences representing genera that have been underrepresented in our study were tested; **(B)** Percentage of the retrieved sequences that were correctly assigned to their respective genera, along with the percentage of sequences from the same genus present in the different colonies analysed in the present study.
